# New age constraints for human entry into the Americas on the north Pacific coast

**DOI:** 10.1038/s41598-024-54592-x

**Published:** 2024-02-21

**Authors:** Martina L. Steffen

**Affiliations:** School of Archaeology, University of Oxford, Oxford, OX1 3TG UK

**Keywords:** Biogeography, Climate-change ecology

## Abstract

The timing of the initial peopling of the Americas is unresolved. Because the archaeological record necessitates discussion of human entry from Beringia into southern North America during the last glaciation, addressing this problem routinely involves evaluating environmental parameters then targeting areas suitable for human settlement. Vertebrate remains indicate landscape quality and are a key dataset for assessing coastal migration theories and the viability of coastal routes. Here, radiocarbon dates on vertebrate specimens and archaeological sites are calibrated to document species occurrences and the ages of human settlements across the western expansion and decay of the Cordilleran Ice Sheet (CIS) during the Late Wisconsin Fraser Glaciation in four subregions of the north Pacific coast of North America. The results show archaeological sites occur after glacial maxima and are generally consistent with the age of other securely dated earliest sites in southern North America. They also highlight gaps in the vertebrate chronologies around CIS maxima in each of the subregions that point to species redistributions and extirpations and signal times of low potential for human settlement and subsistence in a key portion of the proposed coastal migration route. This study, therefore, defines new age constraints for human coastal migration theories in the peopling of the Americas debate.

## Introduction

The peopling of the Americas is an enduring focus of archaeological scholarship and debate^1–38^. The timing of initial human arrivals and the routes people followed from Beringia are not yet fully resolved. Earliest securely dated archaeological sites indicate humans were in or had migrated through southern North America by 14.5 ka to 15.5^39–42^ and around 16 ka ago^43,44^. These sites postdate the Last Glacial Maximum (LGM)^45^ when the Cordilleran Ice Sheet (CIS) and the Laurentide Ice Sheet (LIS) coalesced, blocking mid-continental terrestrial passage south. Current research indicates that a corridor between the decaying ice sheets may have been open from as early about 16 ka–15.5 ka^46^ or 14.9 ka ago^47,48^, although it may not have been fully open before 13.8 ± 0.5 ka^46^ and able to support human life until 13.5–13 ka^49,50^ or as late as 12.7 ka ago^51^. Thus, it is not yet clear if human groups entering North America some 16–14.5 ka ago would have been able to do so via a mid-continental route through an emergent ice-free corridor^34^. An alternative hypothesis suggests these early groups migrated on a north Pacific coast route that was already substantially open for human passage in front of the western margin of the CIS^4,11,15,30,34,35,37,52,53^.

The recent discovery of human footprints in the Lake Otero playa at White Sands National Park, New Mexico is significant in the peopling of the Americas debate. The footprint trackways have been estimated to be 23 ka and 21 ka old based on radiocarbon dates on propagules of the grass-like perennial herb, *Ruppia cirrhosa*, embedded in layers of sediment between the footprints^54^. Controversy about these dates focuses on the possibility of a radiocarbon reservoir in the water in which the dated seeds grew and the sedimentary context of the trackways^55–61^. To address the dating, three new radiocarbon ages on bulk terrestrial pollens from the same sediment layers as those of the *Ruppia* seeds and optically stimulated luminescence (OSL) ages on the sediments from the human footprint–bearing sediment sequence were analysed^62^. The pollen ages span 25.9–20.3 ka ago and include large uncertainties ranging from 2.3 ka to 2.5 ka^54^ characterised the sediments from which the pollen was sampled as alluvial and aeolian. Such sediments consist of redeposited materials and may include ancient pollen that had been eroded and transported from older deposits in the lake basin. Because of this the new pollen dates should be considered maximum ages. Technical descriptions of dated pollens and of the sediments from which they were sampled were not provided and could assist in clarifying the sedimentary context of the radiocarbon dated pollen. The bulk aliquot OSL samples were collected from dark gray clays that are a lithological unit separate from and underlying the deposits that contain the trackways^62^. The Law of Superposition predicts that the trackways must be younger, by some unknown amount, than the 16.2–23.3 ka ages from the OSL results. Therefore, the new dates may generally correspond with the initial seed ages and represent progress in the study of the formation history of the trackways, but do not fully resolve the controversy. Although additional work is needed to assess the age of the White Sands footprints, the potentially early age of the site raises new questions about initial human migrations during the middle of the LGM, when the CIS and LIS restricted human passage southward. The implication being that either humans entered southern North America by land before the ice sheets merged, or they did so along the north Pacific coast at an earlier date than has yet been considered in detail.

**Figure 1:**
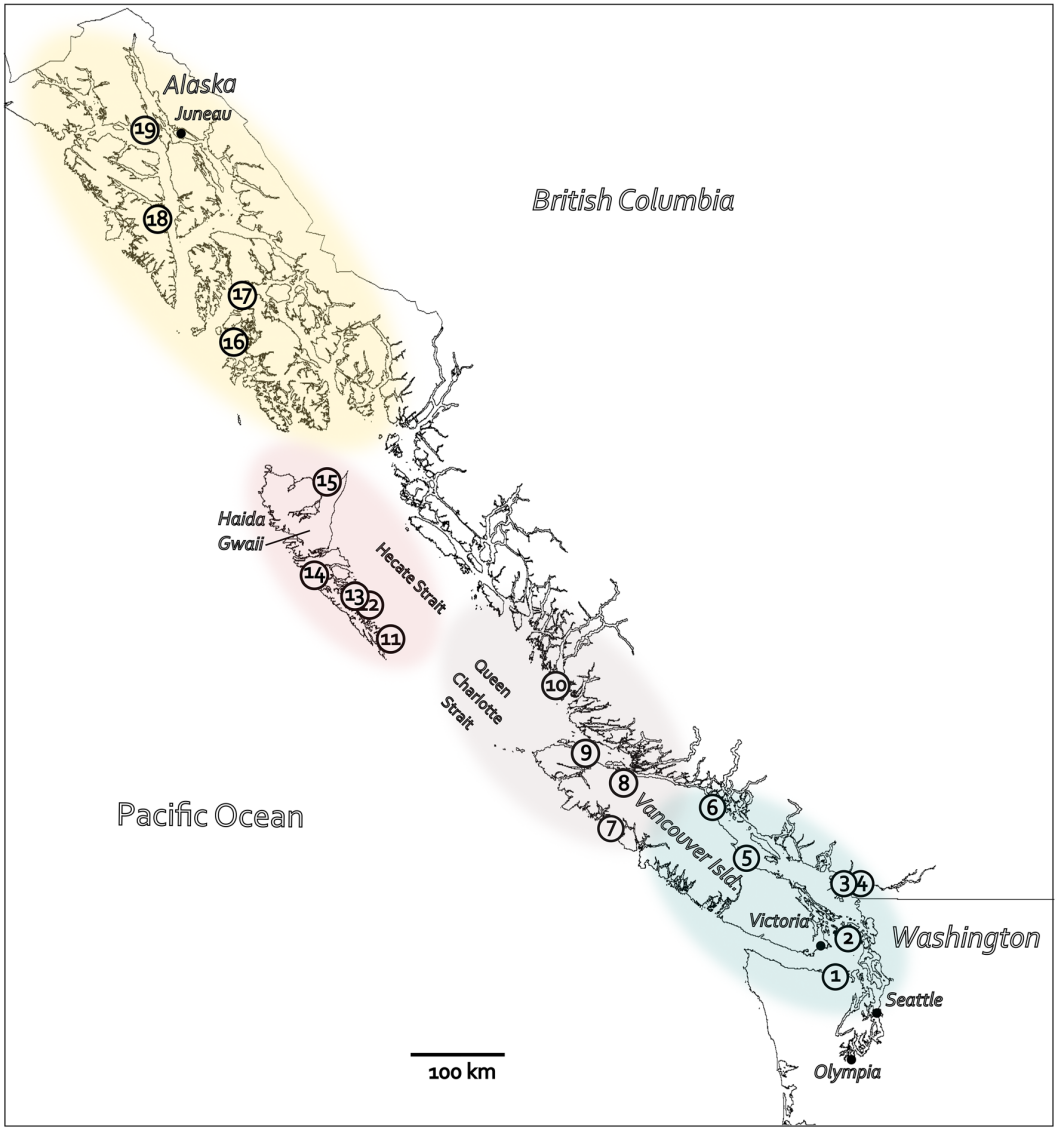
The north Pacific coast showing numbered locations from which radiocarbon dates were included in this study: 1 Manis, 2 Ayer, 3 Glenrose Cannery, 4 Stave Watershed, 5 Courtney, 6 Quadra Island, 7 Port Eliza Cave, 8 P2, Arch-2, Resonance and Sparkle caves and Kokish River, 9 Bear Cove, 10 Namu and Calvert, Triquet and Hunter islands, 11 Kilgii Gwaii, 12 Gaadu Din caves, 13 Richardson Island, 14 K1 Cave, 15 White Creek, 16 Chuck Lake, 17 Alexander Archipelago, 18 Hidden Falls, 19 Ground Hog Bay. Location descriptions are a supplementary file. Subregions: Southeast Alaska—gold, Haida Gwaii—rose, central coast—violet, south coast—blue. (Created in Inkscape 1.2.1. https://inkscape.org).

During the Late Wisconsin Fraser Glaciation on the north Pacific coast, the CIS would have obstructed human settlement and limited subsistence resources where vertebrates declined or were absent due to climate extremes. Although the occurrence and quality of unglaciated areas available for humans has been a central consideration in coastal migration theory since inception^4^, the ecological viability of a coastal route has not been fully evaluated across the western expansion and retreat of the CIS. To address this, the current study assembles radiocarbon dates on vertebrate specimens and archaeological sites from across the north Pacific coast (Fig. 1) and uses current statistical methods to calibrate and model age estimates into chronologies. This approach aims to identify evidence of settings viable for vertebrate and human populations across the growth and decay of CIS. Four subregions of the north Pacific coast are considered: Southeast Alaska, Haida Gwaii, the central coast including north Vancouver Island, and a south coast subregion including southern Vancouver Island and coastal northwest Washington. Each subregion has a specific glaciation history within the broader record^63^ and history of archaeological and paleontological research. The timespan of interest is from 30 ka ago at the transition to the Late Wisconsin as climate changed from interstadial to full glacial through the beginning of the present interglaciation to about 10 ka ago. Results evidence when and where human groups could have been sustained with implications for human coastal migration theory.

## Results

To find evidence of terrain able to support humans across the expansion and decay of the CIS during Late Wisconsin Fraser Glaciation on the north Pacific coast of North America, I constructed four chronologies based on 233 radiocarbon dates from archaeological sites and vertebrate specimens. Table S1 lists each date and its source reference. The ages of archaeological sites are direct evidence of a human presence. Those of vertebrates are proxy records that indicate environments able to provision human populations. Radiocarbon dates were calibrated in OxCal 4.4.4^64^ with IntCal20^65^ and Marine20^66^ data.

**Figure 2:**
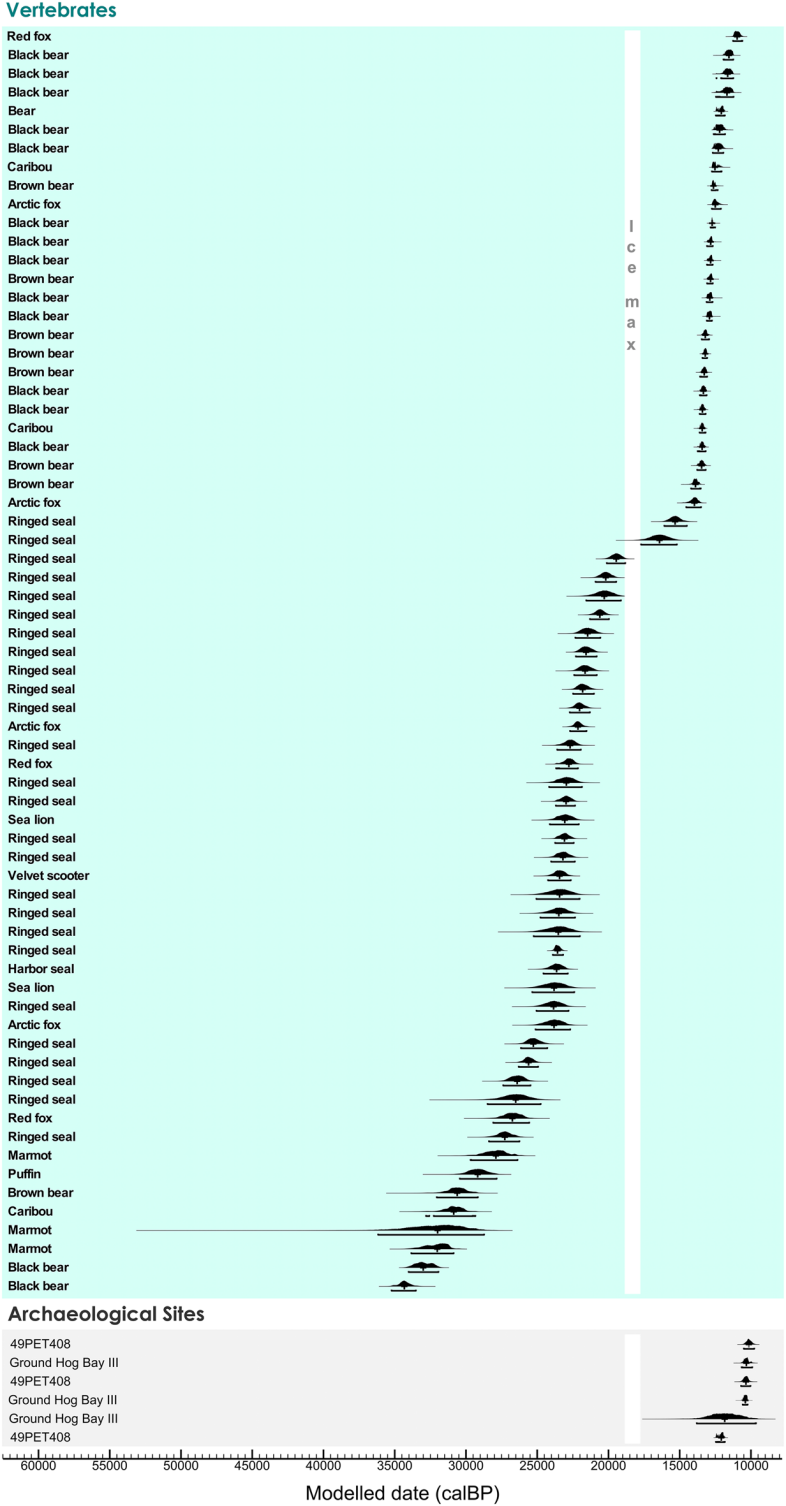
Southeast Alaska chronology. Bayesian model of 74 calibrated AMS ^14^C ages on vertebrates from 15 caves and on bone and charcoal from three archaeological sites in Alexander Archipelago. Each curve indicates a single sample distribution. Calibration and Bayesian modeling are based on OxCal v 4.4.4^64^. The white vertical band indicates the CIS ice maximum and unlikely human settlement.

From Southeast Alaska, 74 radiocarbon dates on bones from 15 caves and three archaeological sites in the Alexander Archipelago were recalibrated and produced a well-constrained chronology (Fig. 2, Fig. S1; Tables S1 and S2). A decline in the number of dates between 20–13 ka ago and a hiatus from at least 17.7–18.82 ka calBP indicates that vertebrates declined in abundance and may have been absent for some 1,120 years due to climate intolerance during the ice maximum. Archaeological sites in Southeast Alaska postdate that maximum and span from 12.08 ka calBP (12.46–11.83 ka calBP, 95.4%).

**Figure 3:**
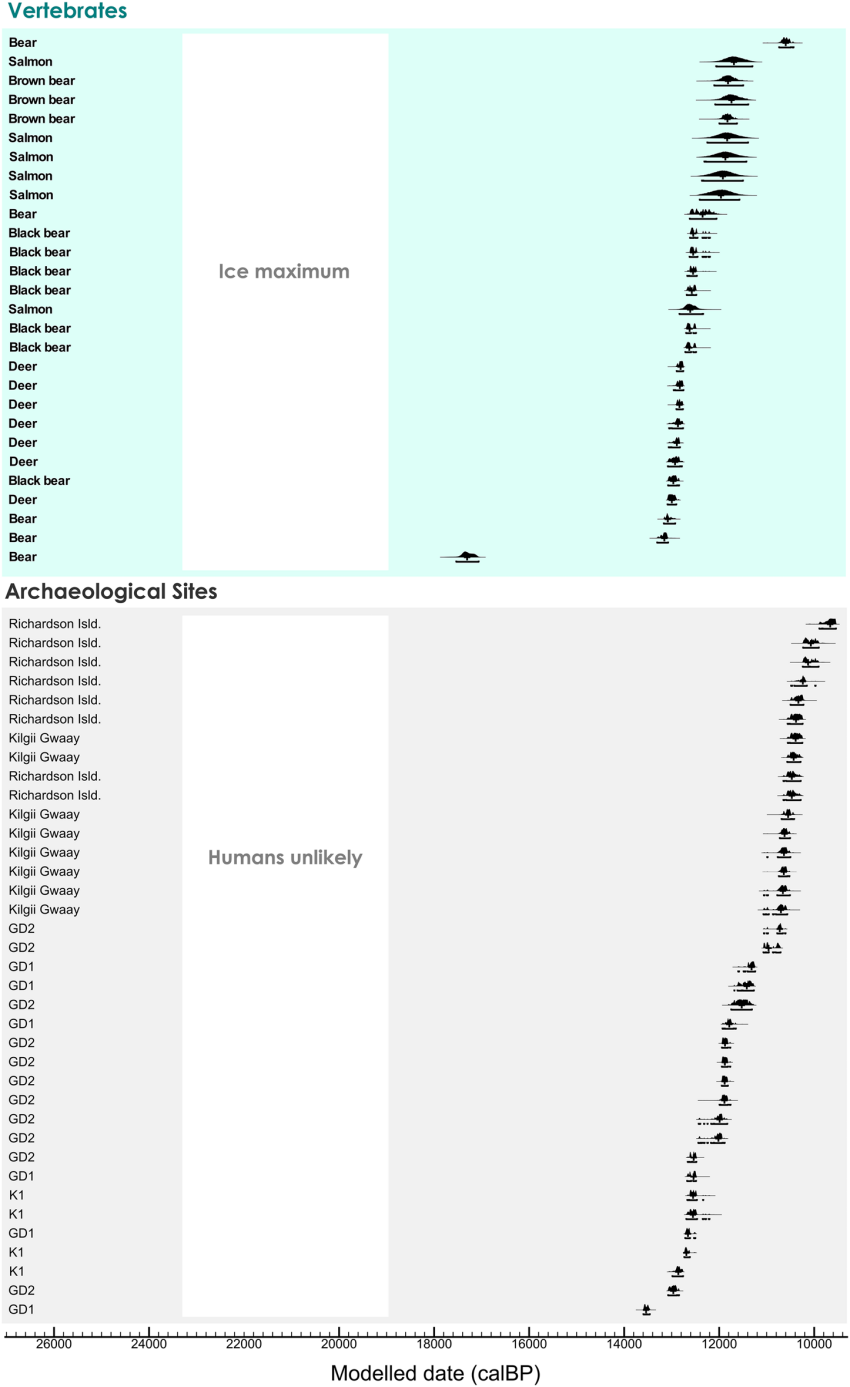
Haida Gwaii chronology. Bayesian model of 66 calibrated AMS ^14^C ages on vertebrates and on bone and charcoal from archaeological sites on Haida Gwaii. Each curve indicates a single sample distribution. Calibration and Bayesian modeling are based on OxCal v 4.4.4^64^. The white vertical band indicates the CIS ice maximum and unlikely human settlement.

The radiocarbon chronology for Haida Gwaii consists of 66 radiocarbon dates on bone and charcoal from six localities (Fig. 3, Fig. S2; Tables S1 and S3). The vertebrate record preceding the Late Wisconsin glacial ice maximum is limited to one date on caribou antler of 45.67 ka calBP (46.99–44.6 ka calBP, 95.4%; not shown in Fig. 3). Remaining radiocarbon dates span the latest Pleistocene and early Holocene. A radiocarbon age estimate of 17.31 ka calBP (17.54–17.06 ka calBP, 95.4%) on a bear bone is the earliest date following the ice maximum. Environmental conditions attracted this large terrestrial mammal. A gap of about 3.8 ka until 13.15 ka calBP (13.31–13.08 ka calBP, 95.4%) suggests bears may have recolonized Haida Gwaii near the onset of the Younger Dryas cold event and then persisted. Earliest archaeological evidence dates to 13.54 ka calBP (13.6–13.46 ka calBP, 95.4%) and continues across several sites into the Holocene.

**Figure 4:**
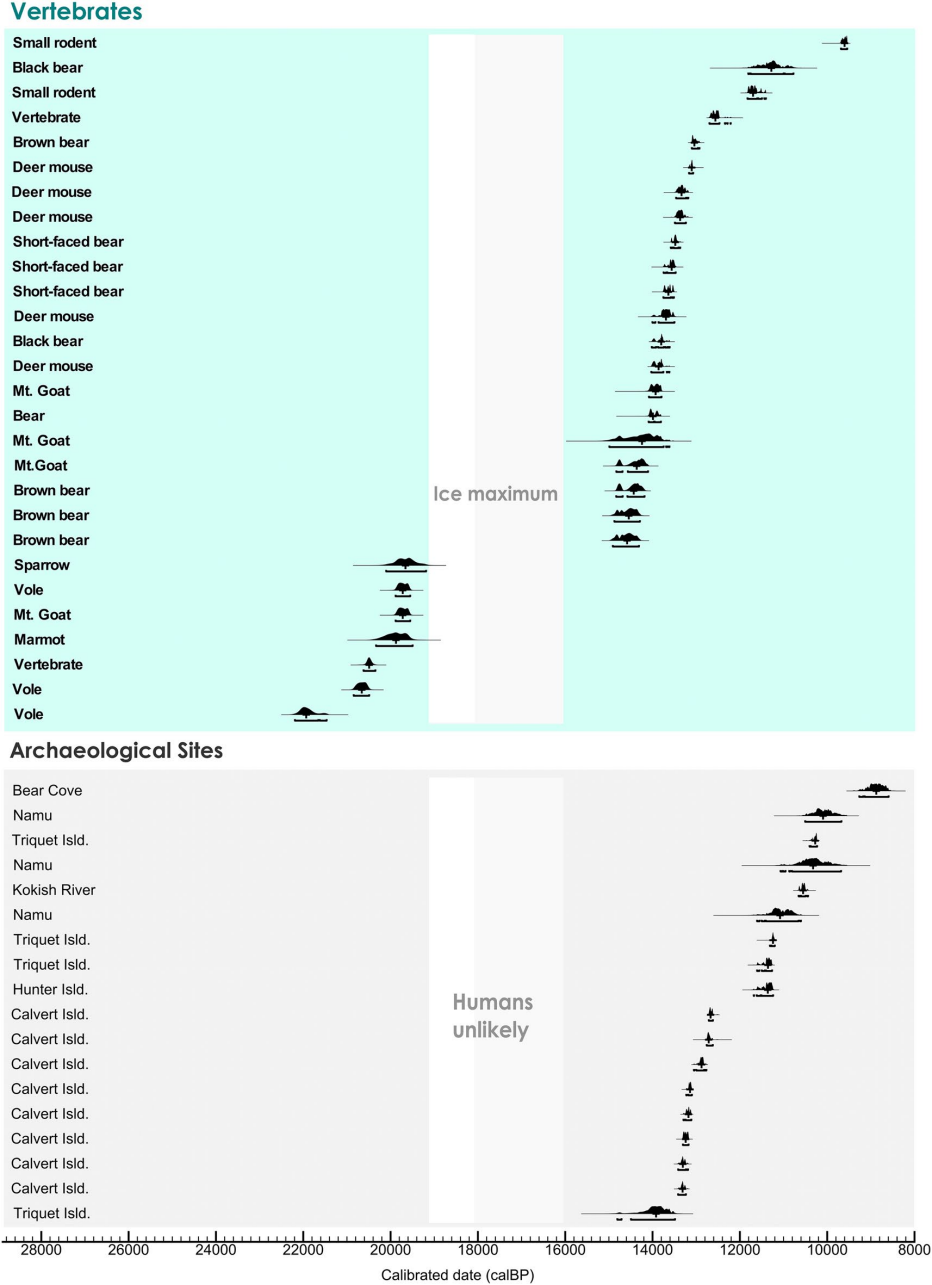
Central coast chronology. Bayesian model of 46 calibrated AMS ^14^C ages on vertebrates and on bone and charcoal from archaeological sites in the central coast subregion of the north Pacific coast. Each curve indicates a single sample distribution. Calibration and Bayesian modeling are based on OxCal v 4.4.4^64^. The white vertical band indicates the CIS maximum in the Queen Charlotte Sound and north Vancouver Island, and the light grey vertical band indicates ice maximum at north Vancouver Island (see manuscript text).

The central cost radiocarbon chronology consists of 46 radiocarbon dates on vertebrates and archaeological materials (Fig. 4, Fig. S3; Tables S1 and S4). The vertebrate record directly preceding the glacial ice maximum comprises a diverse cool temperate fauna from 21.93 ka calBP (22.19–21.46 ka calBP, 95.4%) to 19.66 ka calBP (20.10–19.19 ka calBP, 95.4%). At Port Eliza Cave^67^, a 4.3 ka hiatus in the vertebrate record and a sedimentary deposit indicative of glacial ice cover to 14.36 ka calBP (14.83–14.1 ka calBP, 95.4%) give way to overlying diamict and the return of cool temperate adapted mountain goat and deer mouse. On northeast Vancouver Island, brown bear from 14.58 ka calBP (14.91–14.31 ka calBP, 95.4%) and black bear from 13.8 ka calBP (14.02–13.61 ka calBP, 95.4%) are early colonizers, as is deer mouse at 13.86 ka calBP (14.03–13.62 ka calBP, 95.4%). Archaeological sites span from 13.92 ka calBP (14.81–13.49 ka calBP, 95.4%) and 13.31 ka calBP (13.42–13.24 ka calBP, 95.4%) in the central coast subregion north of Vancouver Island.

**Figure 5:**
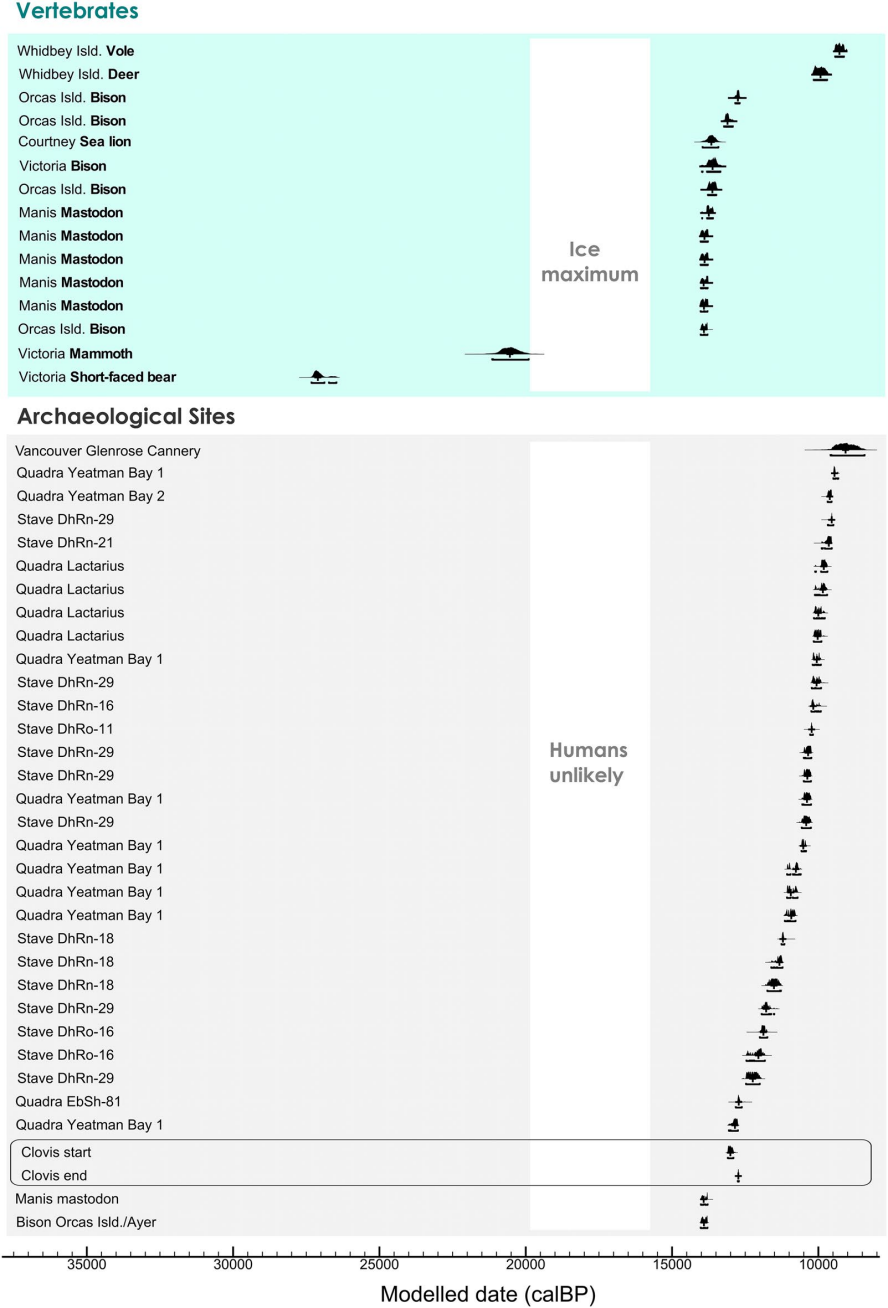
South coast chronology. Bayesian model of 47 calibrated AMS ^14^C ages on bones of vertebrates and on bone and charcoal from archaeological sites in the south coast subregion of the north Pacific coast. Each curve indicates a single sample distribution. Clovis age ranges from^19^ are included as a reference (see text). Calibration and Bayesian modeling are based on OxCal v 4.4.4^64^. The white vertical band indicates the CIS ice maximum and unlikely human settlement.

The radiocarbon chronology for the south coast subregion consists of 47 radiocarbon dates on vertebrates and includes several archaeological sites (Fig. 5, Fig. S4; Table S1 and S5). An age estimate of 20.54 ka calBP (21.15–19.9 ka calBP, 95.4%) on a mammoth bone is the terminal date preceding Late Wisconsin Fraser glacial ice cover on south Vancouver Island. Stellar sea lion is an early colonizer on southeastern Vancouver Island from 13.66 ka calBP (13.955–13.418 ka calBP, 95.4%). Two Pleistocene megafauna species, mastodon and bison, were found with earliest archaeological records at 13.92 ka calBP (14.02–13.79 ka calBP, 95.4%) from Manis^68,69^ (mastodon) and 13.91 ka calBP (14.02–13.79 ka calBP. 95.4%) from Ayer^70^ (bison). Clovis, a distinctive and widespread early North American archaeological culture significant in the peopling of the Americas scholarship, is included as a reference point for the discussion. Clovis age parameters are based on radiocarbon dated sites primarily in the American Southwest and Great Plains^17^ where this culture spans approximately 13.59–12.74 ka calBP (Table S1 and S5).

## Discussion

Occurrences of vertebrates and archaeological sites across the western expansion and retreat of the CIS during the Late Wisconsin Fraser Glaciation define age constraints for human migrations on the north Pacific coast of North America. The CIS had started to develop by 30 ka ago (30–25 ka ^14^C BP)^63^. At its maximum extent ice covered nearly all of British Columbia, southern and central Yukon, and parts of Alaska, Alberta, Washington, Idaho, and Montana^47,48^. It advanced west toward the edge of the British Columbia continental shelf 19.3–20.5 ka ago (16–17 ^14^C BP) and reached maximum southern extent 18.3–17 ka calBP years ago (15–14 ka ^14^C BP), then decayed rapidly due both to climate warming and calving at the western margin of the ice sheet and persisted until 12.9–12.5 ka ago (11.0–10.5 ka ^14^C BP)^71^. The CIS growth and decay and the geometry of its western margin varied across the Pacific coast. Differences in CIS loading and unloading resulted in varied local isostatic responses and sea-level curves^71,72^.

As the CIS expanded west and south, terrain was overrun, and faunal communities were redistributed. Diverse biota of plants and animals persisted beyond glacial ice margins to the north in Alaska and the Yukon, to the south in the northwestern United States, and possibly also in isolated coastal refugia^73,74^.

**Figure 6:**
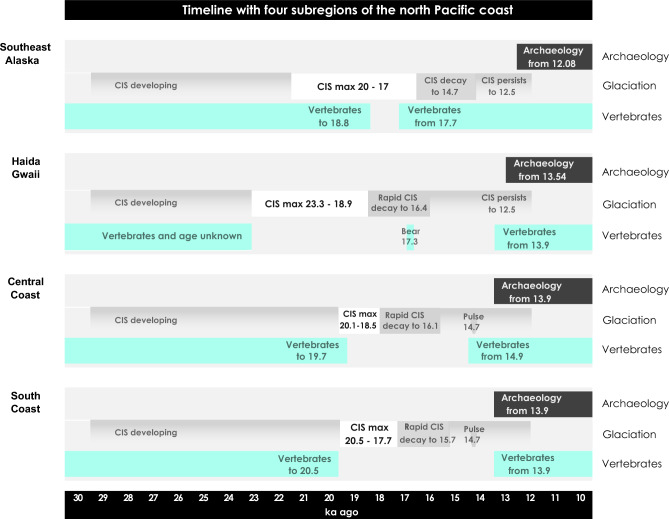
Timeline. Timelines for archaeology, glaciation, and vertebrate occurrences in four subregions of the north Pacific coast: Southeast Alaska, Haida Gwaii, central coast, south coast.

Each of the four north Pacific coast radiocarbon chronologies includes a hiatus when no vertebrates occur and that generally corresponds with the timing of subregional glacial ice maxima (Fig. 6). Reduced numbers of vertebrates or their true absence in extreme climate conditions would have limited or excluded human subsistence. The results of this study do not document direct evidence glacial refugia able to support animals and humans spanning the western expansion and decay of the CIS. Instead, they point to areas habitable by animals and human groups before and after glacial ice maxima and establish new age constraints for coastal migration theories of human entry into the Americas on a north Pacific coast route.

### Southeast Alaska

In Southeast Alaska, the CIS extended along major fjords and reached the western edge continental shelf in some locations^75,76^. Ice flow onto subaerially exposed areas of the inner shelf may have left some ice-free areas^76^, including land now submerged by post-glacial sea level rise and portions of the currently terrestrial land base. These terrestrial areas were assessed in studies that recovered vertebrate remains and archaeological materials^77,78^. An ice sheet chronology was then constructed based on nine cosmogenic ^10^Be exposure-dated rock surfaces at previously mapped terrestrial sites. These averaged 17 ka ± 700 calBP and indicated refugia were not continuously exposed but were covered by ice during the CIS maximum^79^. A recalibration of the radiocarbon date series in^77^ further suggested that ice advanced over Prince of Wales Island between about 19.8 ka calBP and 17.2 ka calBP and with the ice sheet chronology placed the western extent of the CIS in Southeast Alaska between about 20–17 ka ago^79^.

Using current methods, this study recalibrated a Southeast Alaska vertebrate date series^77^ and found the hiatus spanned 18.82 ka calBP and 17.7 ka calBP, which is no less than 1,120 years (Fig. 2). This gap marks the maximum ice extent, a real or near absence of vertebrates, and low potential for human subsistence, migration, and settlement in Southeast Alaska.

The earliest archaeological site in this subregion is Shuká Káa / On Your Knees Cave that dates to about 12.08 ka calBP^78^. Any earlier undocumented human arrivals would have found a nearly continuous availability of animal resources that could have afforded diverse subsistence opportunities. Areas of the continental shelf that may have been subaerially exposed due to changes in local relative sea level during the Late Wisconsin remain unexplored for archaeological or biological evidence of refugia.

### Haida Gwaii

The radiocarbon record on Haida Gwaii follows glacial ice decay. Based on radiocarbon dated plant remains at Mary Point there was a lowland area approximately 80 kms long near the east coast of Moresby Island called the Hecate Refugium that remained ice-free between about 27.96 ka calBP (23.74 ka ± 300 ^14^C BP) and 23.27 ka calBP (19.27 ka ± 360 ^14^C BP)^73^. The CIS maximum occurred in the Haida Gwaii subregion between about 23.3 ka calBP and 19 ka calBP^74,80^, and Haida Gwaii was largely ice free by 18.8 ka calBP (15.5 ka ^14^C BP)^47,48^. Although no continuously unglaciated area spanning the CIS maximum has been documented, small refugia as nunataks, on headlands and inter-fjord ridges, or as now submerged areas in western Hecate Strait may have remained unglaciated^74^. By 15.6 ka ago (13 ka ^14^C BP) the adjacent mainland at Prince Rupert was ice free^63^.

An ursid bone dated to 17.3 ka years ago (14.54 ka ± 70 ^14^C BP) is an earliest post-ice vertebrate record. The near lack of vertebrate remains and radiocarbon dates for Haida Gwaii before glaciation and immediately following ice decay until about 13.3 ka ago indicates either that the environment did not support continuous occupation, that the preservation of animal bones is limited, or that there is a gap in research. Karst caves on Haida Gwaii have been systematically targeted in archaeological surveys that produced faunal specimens and archaeological materials^35,81–84^, which contradicts a gap in research. Sediments removed with glacial meltwater pulses or other preservation issues may explain a near lack of vertebrate remains in karst caves both before and immediately after the CIS maximum. If sediments were removed with glacial meltwater, the radiocarbon record from 13.3 ka calBP may represent a post-glacial stabilization of terrestrial terrain conducive to continuous habitation by animals and humans.

Initial human occupation of Haida Gwaii occurred at Gaduu Din 1 Cave from about 13.54 ka calBP and approximately corresponds with a vertebrate record that includes ursids, ungulates, and salmon as available human food species^82–84^. This suggests that on Haida Gwaii, as in Southeast Alaska, humans populated an ecologically rich environment millennia after the CIS decayed. Haida Gwaii probably was available for migrating humans as ice advanced before about 23.3 ka ago and as ice decayed after 18.8 ka ago, although may not have been viable for human habitation and subsistence following deglaciation until 17.3 ka ago or later.

### Central Coast

Glaciomarine sedimentation in marine core MD02-2496 obtained west of central Vancouver Island signals an initial advance of the CIS at about 30 ka ago (25.6 ka ^14^C BP)^85^. Ice progressed onto the continental shelf proximal to the core after 20.1 ka calBP (16.7 ka ^14^C BP) and was thickest on northern Vancouver Island after ca. 19.2 ka ago (16 ka ^14^C BP)^63^. A ^10^Be dating chronology of deglaciation in the Queen Charlotte Sound area north of Vancouver Island indicated that the western margin of the CIS was retreating there by 18.1 ± 0.2 ka ago and low altitude terrain was ice free by 17.7 ± 0.3 ka ago^86^. Sediments and plant debris show that deglaciation had commenced on northeast Vancouver Island as early as 16.49 ka ago (13.63 ± 0.31 ka ^14^C BP)^87,88^. Increased sedimentation rates resulting from the deposition of ice-rafted debris in marine core PAR85-01 retrieved near southwest Queen Charlotte Sound suggests rapid deglaciation commenced from about 18.87 ka calBP (15.57 ± 0.17 ka ^14^C BP) and continued to 16.43 ka ago (13.59 ± 0.2 ka ^14^C BP)^80^. Deposition of detritus in marine core MD02-2496 from about 17 ka calBP (14 ka ^14^C BP) abruptly terminates 16.09 ka ago (13.5 ka ^14^C BP) signalling the cessation of rapid ice sheet retreat from the continental shelf west of Vancouver Island. A saw-toothed pattern of deposition in that core records smaller pulses of ice expansion and decay that include a notable event at 14.7 ka ago (12.5 ka ^14^C BP) coincident with a possible Oldest Dryas ice readvance and massive ice sheet unloading evidenced in extremely rapid isostatic response^72,85,89^. The CIS persisted until about 12.5 ka ago (10.5 ka ^14^C BP)^63^.

A diverse faunal community was present during the CIS ice advance on northwest Vancouver Island and until 19.2 ka ago^67^. As the CIS expanded westward and covered Vancouver Island, vertebrate faunas perished or relocated to unglaciated areas. Vertebrates recolonized northeast Vancouver Island as early as about 14.9 ka ago^90,91^ and northwest Vancouver Island by about 14.36 ka ago^92^. Several species including mountain goats^93^, deer mice^94^, and brown bears^90^ probably arrived on Vancouver Island from south of the CIS margin.

A gap in the vertebrate record of the central coast from about 19.7 ka calBP to 14.9 ka calBP is generally consistent with geological indicators of maximum glacial ice extent and extends well into the span of ice decay. The earliest post-ice vertebrate record occurs some 2 ka after the western margin of the CIS had retreated significantly exposing low altitude terrain. This may signal a period of biotic development and delayed animal recolonizations that could have limited human food resources. Archaeological sites in the central coast subregion currently date to between 14–11.5 ka ago^35^ in a developed marine and terrestrial biotic setting.

Future archaeological studies on the central coast could identify and target low altitude terrain that was ice free by about 18 ka. Additionally, grounded glaciers might only have reached part way across the continental shelf^95,96^ and portions of the shelf including Goose, Cook and Middle banks might have been terrestrial landforms or shoals when the CIS was proximal^97^, affording any migrating human groups access to watercraft landing areas as well as nearshore marine foods and any surviving terrestrial resources. These now-submerged areas have not yet been explored for archaeological evidence.

### South Coast

In the south coast subregion, the CIS thickened to the extent that it covered the Vancouver Island Mountains^63^. The CIS advanced west onto the continental shelf about 19.3–20.5 ka ago (16–17 ka ^14^C BP), reached maximum southern extent in the Puget Lowland at about 17.7 ka ago (14.5 ka ^14^C BP)^98^, and then retreated. Ice decay occurred rapidly as frontal retreat and downwasting^98^ and caused marine incursion into Juan De Fuca Strait 16.3 ka ago (13.6 ka ^14^C BP)^85^. Vancouver and Victoria were ice free by 15.7 ka ago (13.1 ka ^14^C BP), and glaciers had largely disappeared from this subregion by 12.5 ka ago (10 ka ^14^C BP)^63^.

Near the onset of the Late Wisconsin Fraser Glaciation, vertebrates on the south coast included extinct Pleistocene species in parkland and cold steppe grassland environments^99^. After 20.5 ka calBP, a gap in the vertebrate record corresponds with the CIS maximum. Stellar sea lions had colonized waters near Courtney at 13.66 ka calBP^100^. By 13.9 ka calBP bison were on southern Vancouver Island and the San Juan Islands^101^, and mastodon were on the Olympic Peninsula at Sequim^68,69^. Archaeological evidence of human hunting appears to occur directly with mastodon bones at 13.92 ka calBP at Manis^68,69^ and on bison bones at 13.91 ka calBP from the Ayer^70^. This suggests that human subsistence relied at least in part on large terrestrial animals in this period on the south coast. It is unclear if Manis and Ayer predate the Clovis archaeological culture or not because there are no directly and securely dated Clovis sites in the Pacific Northwest with which to compare them. Clovis^92,102^ and Clovis-style^103^ artifacts as surface finds and Clovis artifacts at the East Wenatchee site are not directly dated^104,105^. Clovis in the study area could be slightly younger or older than the established 13.59–12.74 ka calBP Clovis age range.

Human migration and settlement are least likely during CIS ice cover from 20.5 ka calBP to about 17.7 ka calBP and subsistence resources may have been limited as glacial ice decayed to as late as around 13.9 ka calBP.

### Coastal Migration

Human migrations into the Americas along the north Pacific coast would have depended on a route without physical barriers and the availability of subsistence resources. The western expansion and retreat of the CIS largely controlled the amount and quality of habitable terrain available for any human groups along the coast. Because boat-based human coastal migration required periodic landfall that would have been obstructed by glaciers, the existence of a chain of ice-free refugia to host human migration during the last glacial is integral to coastal migration theories^4,30,37^. The availability of subsistence and food sources would have been essential and may have included terrestrial resources or been fully marine^15,37,83^.

This study found no evidence in the vertebrate and archaeological records of refugia spanning the entire CIS western expansion and retreat on the north Pacific coast. Instead, two main constraints on human migrations are highlighted: (1) the CIS maximum was a physical barrier to migration that denotes when human migrations are unlikely to have occurred, and (2) an apparent decline or absence of vertebrates may signal times of reduced biotic activity and limited human subsistence resources immediately before, during and for hundreds of years and longer durations after CIS maxima. The current archaeological record in the study area occurs after CIS maxima, near the end of glacial ice decay, and is consistant with these constraints.

To firmly establish the north Pacific coast as a route by which humans are likely to have migrated from Beringia into southern North America, archaeological sites on the coast should pre-date those to the south. The current archaeological record starts approximately 14 ka ago. This is somewhat later than although generally consistent with the age of earliest securely dated archaeological sites south of the ice sheets in the 16–14.5 ka old range. Future archaeological research on the north Pacific coast may well discover other early sites that post-date glacial ice maxima and are in the 18–15 ka age range.

If a 21–23 ka old record of human footprints at White Sands is confirmed or other sites of a similar age are found, archaeological evidence on the north Pacific coast that matches or pre-dates the record to the south will be needed to strongly support costal migration theory and a Pacific coastal route from Beringia into southern North America. Toward that end, this study shows that coastal terrain hosted vertebrates and could have been accessed by human groups before CIS ice cover in Southeast Alaska 18.8 ka ago, on Haida Gwaii likely prior to about 23.3 ka ago, and in the central and south coast subregions before 19.7 ka ago.

## Conclusions

The ages of vertebrate specimens and archaeological sites signal environments suitable for human subsistence and settlement across the Late Wisconsin expansion and decay of the CIS on the north Pacific coast of North America. Four radiocarbon date series reveal gaps during CIS western ice maxima when human groups are least likely to have migrated or settled. In Southeast Alaska, the vertebrate record is nearly continuous with a hiatus from about 17.7–18.82 ka calBP at an ice maximum. On Haida Gwaii, a gap in the vertebrate record corresponds with ice extent from around 23–19 ka and then persists to 17.31 ka calBP and 13.3 ka calBP, which may point to periodic vertebrate colonisations. On the central coast, the ice maximum spans from about 20.1–18.5 ka calBP. A hiatus in the vertebrate record occurs from 19.2–14.9 ka calBP on north Vancouver Island and animals and humans occur as early as 13.9 ka ago on Triquet and Calvert islands. On the south coast, a gap in the vertebrate record occurs during the ice maximum from 20.5–17.7 ka calBP and continues to about 13.9 ka calBP when humans and vertebrates co-occur. This record indicates that a corridor in front of the expanding CIS would have been substantially open for human migrations along the north Pacific coast during the Late Wisconsin until about 23.3 and 20 ka years ago, after which glacial ice cover probably hindered migrations until about 18.9 ka and 17.7 or 17 ka years ago. A coastal route is likely to have been at least partially open during initially rapid CIS decay and gaps in the vertebrate record to as late as about 14 ka years ago suggest reduced biotic activity may have challenged human subsistence. These age ranges define physical and subsistence constraints on human migrations for coastal migration theories in the peopling of the Americas debate.

## Methods

The 233 radiocarbon dates on vertebrate specimens analysed in this study are from published sources (Table S1). These originate from several laboratories that are indicated by the sample name and number. Laboratory naming conventions are as follows: AA—University of Arizona Accelerator Mass Spectrometry (AMS) Laboratory, USA; Beta—Beta Analytic, USA; CAMS—Center for Accelerator Mass Spectrometry, Lawrence Livermore National Laboratory, USA; GaK—Gakushūin University, Japan; GX—Geochron Laboratories, Inc., Cambridge, Massachusetts, USA; OxA—Oxford Radiocarbon Accelerator Unit, University of Oxford, UK; TO—IsoTrace Laboratory, University of Toronto, Canada; UCIAMS—Keck-CCAMS Group, Irvine, California, USA; UOC—A.E. Lalonde AMS Laboratory, University of Ottawa, Canada; WAT—University of Waterloo, Canada; and WSU—Washington State University, USA.

OxCal v 4.4.4 was used to calculate calendar age estimates from radiocarbon age determinations and for probabilistic Bayesian modeling of calibrated ^14^C determinations following^64^. Radiocarbon age determinations reported previously were recalibrated. Age estimates on terrestrial samples were calibrated based on atmospheric data from IntCal20^65^. Those on marine samples were calibrated based on data from Marine20^66^. Marine reservoir offsets in marine samples were calculated using ΔR values from the 14CHRON Marine20 marine radiocarbon reservoir database at http://calib.org/marine/^106^. The ΔR values in that database have been recalculated to provide a ΔR_20_ for use with Marine20^107^. Marine offset values vary in subregions from south to north along the north Pacific coast^108^. To account for this variation, averaged ΔR values were calculated using published datapoints in the 14CHRON Marine database. A ΔR value of 257 ± 99 based on the weighted average offset of 32 datapoints (Table S6) was applied to marine samples from Southeast Alaska and Haida Gwaii. A ΔR value of 267 ± 52 based on the weighted average offset of 19 datapoints (Table S6) was applied to marine samples from the south coast subregion.

Radiocarbon samples previously identified as mixed marine and terrestrial were assigned a percent marine amount based on δ^13^C and δ^15^N values for each analyzed bone sample (Table S7). I used the FRUITS 3.0 Beta program^109^ to model the proportional contributions of terrestrial and marine food sources to the value of the δ^13^C and δ^15^N isotopes. Where δ ^15^N was not published with a radiocarbon date, I estimated this value based on δ ^15^N values from published sources that best match the spatial and temporal resources available to the mixed feeders in this study to avoid issues associated with baseline shifts and Suess effects^110^. The bone collagen-collagen trophic enrichment factor of δ^13^C 1.0 ± 0.3‰ and δ^15^N 4.2 ± 1.4‰ based on data from archaeological sites^111^ was used.

A radiocarbon date of 14,390 ± 70 (CAMS 75,746) on a bear bone from Haida Gwaii was noted as a mixed feeder^81^. No stable isotope values were reported with this sample. However,^81^ stated that “…an estimated correction of − 150 years has been applied to CAMS 75,746.” To calibrate this date, I added 150 to the reported age of 14,390 ± 70 ^14^C BP and used the age determination 14,540 ± 70 ^14^C BP with ΔR 257 ± 99 and an estimated 30% marine diet in the OxCal calibration model (Table S7).

Radiocarbon ages, ^14^C, are reported as before present, BP. Calibrated radiocarbon age estimates are indicated as calendar years before present, calBP. In both instances, the present age refers to Gregorian years before mid-1950 CE. The abbreviation “ka” indicates thousands of years (kiloannus). The results of calibrations are given as median age estimates and as age ranges that are 95.4% confidence intervals.

### Supplementary Information

Supplementary Information.

## Data Availability

All data, code, and materials used in the analyses are available in the main text or the supplementary materials.
