# Supplementary material for: New age constraints for human entry into the Americas on the north Pacific coast
Source: Sci Rep. 2024 Feb 21;14:4291. doi: 10.1038/s41598-024-54592-x (PMC10881565; doi:10.1038/s41598-024-54592-x)
Supplement: Supplementary file 1 — Supplementary Information. [file 41598_2024_54592_MOESM1_ESM.pdf]

Science Advances

AAAS

Supplementary Materials for

New age constraints for human entry into the Americas on the north Pacific coast

Martina L. Steffen*

*Corresponding author. Email: martinasemail@gmail.com

This PDF file includes:

Supplementary Text
Figures S1 to S4
Tables S1 to S7
References *112–154*

Supplementary Text

This supplementary text lists archaeological and paleontological sites in four subregions of the north Pacific coast – southeast Alaska, Haida Gwaii, central coast, and south coast – from which the radiocarbon dates in this study were derived.

The Sites

Southeast Alaska Subregion –

Limestone caves, Alexander Archipelago. – [*77*] excavated and sampled numerous caves in the Alexander Archipelago including El Capitan, Bumper, Devil's Canopy, Tlacatzinacantli, and Shuká Káa / On Your Knees caves. Vertebrate specimens from this group of sites span the past 50 ka and illustrate a complex faunal history during varied environmental conditions. Our study includes vertebrate specimens directly radiocarbon dated to before, during, and after the CIS

maximum, from approximately 28 ka ^{14}C BP to 10 ka ^{14}C BP (Table S2). The transition to late Wisconsin glacial conditions is evident in a largely cool temperate and cold adapted fauna that includes heather vole, brown lemming, hoary marmot, caribou, saiga, Arctic fox, and brown and black bears, and puffin [*77*]. A primarily sea ice fauna with ringed seal that breed on land-bound sea ice, as well as Steller sea lion, harbour seal, and Arctic and red fox was present closest to the local ice maximum. A faunal transition occurred following ice retreat and into the early Holocene in which cold-adapted species such as caribou, brown bear, wolverine, and tundra vole became extinct locally and the modern forest community including mule deer, black bear, grey wolf, mustelids, and long-tailed vole persisted as a coastal rainforest was established.

On Your Knees Cave / Shuká Káa [49PET408). Archaeological materials from this site on northern Prince of Wales Island are the oldest currently known from southeast Alaska. The archaeological assemblage includes a microblade component, a foliate bifacial projectile point, a flint flaker of mammal bone, a medial fragment of a barbed harpoon, and human remains [*78*, 112]. A radiocarbon age estimate on an awl or punch made of terrestrial mammal bone dates human occupation to 10,300 ± 50 ^{14}C BP [*112*]. A partial human skeleton radiocarbon dated to 9,880 ± 50 ka ^{14}C BP and 9,730 ± 60 ka ^{14}C BP has isotopic values indicative of a fully marine diet. These human skeletal remains are probably contemporaneous with the microblade component at this site and part of the Northwest Coast Microblade Tradition, an American Paleoarctic technology that dates to the early Holocene [*113-115*] and may be related to the earlier Denali Complex in interior Alaska [*112*].

Early Holocene microblade sites. The earliest archaeological sites in southeast Alaska include the early Holocene aged Component 1 at Hidden Falls [*113*], Component III at the Ground Hog Bay 2 site that has been dated from as early as 10,180 ± 800 [*114*], and Locality 1 at Chuck Lake that has an early Holocene component containing several hundred faunal specimens consisting primarily of fish including Salmonidae [*115*]. [*112*] assigned these sites to the Northwest Coast Microblade Tradition.

Haida Gwaii Subregion –

White Creek / SGidlii Gandl, *north Graham Island*. A partial fossil Dawson caribou (*Rangifer tarandus dawsoni*) antler is the only pre-LGM fossil of a large terrestrial vertebrate on Haida Gwaii [*116*]. A radiocarbon date confirmed the age of the specimen as 43,209 ± 649 ^{14}C BP (MIS 3), before the onset of the most recent glaciation. Abundant coprophilous Sporormiella and Sordaria fungal spores were found in peat samples from Cape Ball and indicate the former presence of large herbivores on northeast Haida Gwaii during the middle Wisconsin [*117*].

K1 Cave. – K1 Cave (FgUc-6) is located at 20 m elevation on the west coast of Graham Island. Paleontological and archaeological materials have been found at this site. Sediments consist of pebble to cobble gravels indicative of fluvial conditions that are overlain in some places by silt deposited in aeolian and low velocity aqueous conditions [*81*]. The late Pleistocene paleontological assemblage is dominated by specimens of bear (*Ursus*) and includes mouse (*Peromyscus*) and deer (*Odocoileus*) that may be late Pleistocene or Holocene, as well as domestic dog (*Canis familiaris*) that is late Holocene. A radiocarbon date on a black bear femur indicates the presence of this large terrestrial carnivore at 14,390 ± 70 BP as glacial ice decayed.

Archaeological materials from K1 Cave consist of two projectile point bases associated with black bear remains. One point is constrained between age estimates of 10,660 ± 40 ^{14}C BP and 10,525 ± 50 ^{14}C BP, and the other by dates of 10,960 ± 35 ^{14}C BP BP and 10,510 ± 35 ^{14}C BP [*83*, *84*]. The point fragments probably entered the cave embedded in a hunted black bear and there is no evidence of human habitation at the find location [*82*, *83*]. [*118*] assigned the leaf-shaped biface fragments from K1 Cave to the earliest lithic component on Haida Gwaii, the Kinggi Complex (10.6–8.75 ka ^{14}C BP).

Gaadu Din 1 (1693T). Gaadu Din 1 is a limestone cave at 40 m elevation on Huxley Island, southeast Haida Gwaii. Excavation in a small cavern several meters from the cave entrance produced a paleontological assemblage and archaeological artifacts [*82*]. The paleontological assemblage was primarily in a diamict of gravel, sand, and cobbles and separate from archaeological materials [*83*, *84*]. The late Pleistocene mammalian faunal assemblage includes black bear from ca. 11,030 ± 30 ^{14}C BP and brown bear from 10,715 ± 30 ^{14}C BP to 10,465 ± 30 ^{14}C BP [*83*]. Ungulates including caribou (*Rangifer tarandus*) and deer (*Odocoileus hemionus*) range from 11,060 ± 30 ^{14}C BP to 10,920 ± 35 ^{14}C BP, and a diverse fish assemblage is dominated by salmon (*Oncorhynchus*) that has been dated from 11,510 ± 25 ^{14}C BP to 10,839 ± 25 ^{14}C BP. Overall, the faunal assemblage documents a diverse and abundant fauna from about 11.5 ka ^{14}C BP.

The archaeological assemblage from Gaadu Din 1 Cave includes a bifacial spear point, a spear point base, the tip of a bone point, and two flake tools. The artifacts were from excavated contexts within 10 meters of the cave entrance. The biface is associated with charcoal dates of 9,980 ± 30 ^{14}C BP, the bone point fragment was directly dated to 10,150 ± 25 ^{14}C BP, and the

flake tools are associated with dates of 10,550 ± 25 ^{14}C BP and 10,615 ± 30 ^{14}C BP [*82*, *83*]. The biface base is characteristic of the Kinggi Complex [*82*-*84*]. Gaadu Din 1 Cave was a hunting location and temporary camp [*84*].

Gaadu Din 2 (1906T). Gaadu Din 2 is a small limestone cave located at 100 m elevation about 300 m from the entrance to Gaadu Din 1 [*82*-*84*]. Excavations a few metres inside the cave recovered a lithic assemblage, a small number of fish and bird bones, and a black bear bone. The Kinggi Complex lithic assemblage consists of a spear point, two biface point tips, a bifacial knife, and biface resharpening flakes. Charcoal from 1 cm directly below the spear point gave a date of 10,220 ± 30 ^{14}C BP. The bifacial knife is associated with a charcoal date of 10,295 ± 25 ^{14}C BP. A hearth feature at the cave entrance that shows evidence of reuse and was dated to 9485 ± 15 ^{14}C BP and 9530 ± 15 ^{14}C BP from its upper layer, 10,205 ± 20 ^{14}C BP, 10,210 ± 20 ^{14}C BP, 10,215 ± 20 ^{14}C BP in a middle layer that produced two biface point tips and several biface resharpening flakes, and 10,295 ± 25 ^{14}C BP in the lower layer [*84*]. The cave was a temporary camp.

Richardson Island (1127T). The Richardson Island archaeological site is located on raised marine landforms in North Darwin Sound in the southern Haida Gwaii archipelago. This multicomponent stratified site has been extensively excavated. The first human occupation occurred on a supratidal marine berm dated from 9,290 ± 50 ^{14}C BP [*82*]. Rapid sea level rise resulted in deep and finely stratified deposits until the marine maximum at approximately 8,900 ^{14}C BP. Between about 9.3 ka ^{14}C BP and 8.75 ka ^{14}C BP, the lithic assemblage is dominated by bifacial and unifacial Kinggi Complex tools [*82*]. The unifacial assemblage includes discoidal

and unidirectional core reduction strategies [*84*]. Several bifacial foliate projectile points from this assemblage resemble those found across the north Pacific coast, including Namu and Shuká Káa [*84*]. *Xil* are foliate points similar to those from the Ushki site in Kamchatka where they date from ca. 11,300 to 10,100 BP, and from the Uptar site near Magadan on the Pacific coast of northeast Asia where they underlie an 8,500 BP tephra [*118*]. Kinggi Complex bifacial tools may have been used for terrestrial mammal hunting [*82*, *84*]. At the Richardson Island site, microblades are introduced into the bifacial and unifacial toolkit in a layer dated to 8,750 ± 60 ^{14}C BP, the earliest date for microblade technology on Haida Gwaii [*82*]. As is the case for southeast Alaska, microblade technology on Haida Gwaii may be related to the Denali Paleoarctic Tradition Denali complex from interior Alaska [*112*, *118*]. The faunal assemblage from the Richardson Island site was derived from a series of hearth features excavated from high-resolution stratigraphy and includes numerous fish taxa including salmon, small bird, deer mouse, and mammals. The faunal suite signals that human dietary sources were both marine and terrestrial [*119*].

Kilgii Gwaay (1325T). This intertidal archaeological site is located on a small island in southern Haida Gwaii. The site comprises a surface lag deposit that contained abundant stone tools and a buried shell midden with organic and inorganic cultural materials [*84*]. Radiocarbon dates constrains human occupation at the site to approximately 9.45 ka ^{14}C BP [*84*]. The stone assemblage is primarily unifacial and includes distinctive discoidal cores. A Kinggi Complex lithic assemblage is present at Kilgii Gwaay and at several other less intensively investigated intertidal lithic sites on Haida Gwaii [*120*]. Organic technology from the site includes three-strand twine and wooden hafts and wedges. The faunal assemblage contains more than 30 taxa

that show a marine dietary emphasis on fishes, alcids, albatross, harbour seal, sea mussel, and sea otter. Black bear is also an important component of the assemblage. The faunal remains demonstrate that the early Holocene occupants of southern Haida Gwaii accessed wide range of ecological niches and were reliant on marine resources.

The Central Coast Subregion –

Namu Site (ElSx-1). The Namu archaeological site is in the community of Namu, on the central mainland coast of British Columbia, about 150 kms north of Vancouver Island. Archaeological excavations at Namu were conducted between 1969 and 1994 and exposed a 10-ka old occupation sequence consisting of five periods. The lithic assemblage from the initial occupation in Period 1 dated to 9,720 ± 140 ^{14}C BP includes microliths, core and denticulate scrapers, retouched and utilized flakes, and flaked bifacial points and knives. A marine subsistence orientation is likely to have been present from the date of initial human habitation at this site, although the earliest occupation layers do not contain preserved vertebrate specimens [*121*, *122*]. The overlying archaeological sequence includes marine and terrestrial fauna that indicate reliance on marine species.

Hunter Island (ElTa-18). This shell midden site is located on Hunter Island west of Namu and was investigated during reconnaissance using percussion coring and auger test sampling [*123*]. Charcoal from near the base of the cultural deposit was recovered through percussion coring and radiocarbon dated to 9,940 ± 50 ^{14}C BP. Archaeological investigation has demonstrated that the settlement history of the Hunter Island area is linked to gradual long-term decline in sea level [*123*].

Calvert Island (*EjTa-4*). Calvert Island is in Hakai Passage on the central coast of mainland British Columbia. Sea-level reconstructions and archaeological investigations found ancient shorelines sites on Calvert Island including one site with 29 human footprints in a 13-ka old subsurface paleosol yielding lithic flakes, cores, pebble, flake, and spall tools [*35*, *124*]. Age estimates on pine wood fragments from Stratum X, the paleosol in which the footprints were found, range from 10,720 ± 60 to 11,435 ± 30 [*124*].

Triquet Island (EkTb-9). Triquet Island is located north of Calvert Island in Hakai Passage. Archaeological deposits span from the early post-glacial and show lengthy geoarchaeological and paleoenvironmental sequences as well as lithic and faunal assemblages [*125*]. Lithic tools from the lowermost cultural deposits are dated to as early as 12,010 ± 180 ^{14}C BP and include bi-polar reduction and anvil-rested percussion core and flake tool technologies. The lowermost cultural deposits containing fauna date to as early as 9,140 ± 25 ^{14}C BP and include a diverse marine-based subsistence suite showing an early focus on sea mammals. This site documents long-term land use that links with indigenous oral histories [*125*].

Bear Cove Site (EeSu-8). Bear Cove is a multi-component archaeological site located at Hardy Bay on northeast Vancouver Island [*126*]. The lowermost chipped stone assemblage in Component I is typical of the Pebble Tool Tradition [*127*], an early Holocene component that is widespread in the central and southern north Pacific coast. Component I spans from 8,020 ± 100 ^{14}C BP to about 5,000 years ago [*126*]. The faunal assemblage contained 36% higher percentage of sea mammal, 16% higher percentage of bird, and 32% lower percentage of fish than the

overlying and younger Component III. The early fauna includes dolphin and northern fur seal, rockfish as the highest percentage taxon of fish, mule deer as the dominant terrestrial vertebrate, and ducks and loon in the avian assemblage [*126*].

Kokish River site (*EdSr-42*). This archaeological site consisted of a subsurface lithic assemblage on a raised delta feature [*91*]. It was found during an archaeological overview and impact assessment of a proposed run-of-river hydroelectric project located on the Kokish River, northeastern Vancouver Island. A radiocarbon date from this site is associated with the lithic assemblage that includes several flake tools. Charcoal in close association with these tools gave 9,345 ± 25 ^{14}C BP. The characteristics of the lithic assemblage are consistent with this date.

Pellucidar 2 Cave (P2). P2 Cave is a paleontological site on northeast Vancouver Island at approximately 600 m elevation [*90*, *91*]. Survey and subsurface excavation observed late glacial and early Holocene sediments [*91*]. Radiocarbon age estimates on terrestrial vertebrate bone samples range in age from 8,660 ± 30 ^{14}C BP to 12,440 ± 35 ^{14}C BP. The vertebrate assemblage consists of a few hundred specimens in total including those of short-faced bear, brown bear, mule deer, marmot, tree squirrel, *Microtus* voles, heather vole, deer mouse, shrew, vesper bat, reptiles and amphibians, and birds [*90*, *91*, *128*]. Brown bear is an early post-glacial terrestrial colonizer at 12,440 ± 35 ^{14}C BP and may have competed for resources with short-faced bears arriving by 11,775 ± 30 ^{14}C BP [*90*]. Genetics on the brown bear specimens from P2 Cave suggest these bears colonized the island from the south and east [*90*]. Vertebrates are abundant and diverse from about 10,100 ± 30 ^{14}C BP indicating that complex biotic communities had developed following deglaciation [*128*].

Arch-2 Cave. Arch-2 Cave is a paleontological site located in limestone east of Nimpkish Lake at approximately 600 meters elevation. Systematic excavation recovered an assemblage of some 200 faunal specimens that include Keen's mouse, Vesper bat, shrew, microtine rodents, tree squirrel, brown bear, small birds and a fish. Radiocarbon age estimates on vertebrate specimens range in age from 12,370 ± 35 ^{14}C BP to 11,460 ± 60 ^{14}C BP [*90*, *128*].

Resonance Cave. Resonance Cave is a paleontological site located east of Nimpkish Lake at approximately 800 m elevation. Seven bones of mountain goat (*Oreamnos americanus*) including a femur radiocarbon dated to 12,200 ± 190 ^{14}C BP were recovered from a muddy pool approximately 30 m from the cave entrance [*129*].

Sparkle Cave. Sparkle Cave is a paleontological locality in limestone at approximately 500 m elevation on northeast Vancouver Island nearby and in the same bedrock formation as P2 and Arch-2 caves. It consists of a small opening to a downward trending tube that is entirely blocked with sand and gravel. A partial bear cranium recovered from this deposit was donated to the provincial museum in Victoria, BC. The author examined the specimen and determined its size and characteristics are consistent with late Pleistocene Vancouver Island black bear [see 130]. A radiocarbon date on a fragment of parietal bone yielded an age of 11,935 ± 40 ^{14}C BP [*90*].

Port Eliza Cave. Port Eliza Cave is a sea cave in volcanic bedrock located on northwest Vancouver Island at approximately 85 m elevation [*67*, *92*]. Sedimentological analysis defined strata spanning nonglacial diamict at 18,010 ± 100 ^{14}C BP through 16,270 ± 170 ^{14}C BP, glacial

laminated deposits, and a return to nonglacial conditions at approximately 12,340 ± 50 ^{14}C BP. The pre- glacial maximum fauna contains nine fish species, western toad, at least six bird species, and eight mammalian species including Townsends and long-tailed voles, heather vole, marmot, pine martin, mountain goat and a canid-sized carnivore [*67, 92*]. The post glacial maximum faunal assemblage from this site consists of deer mouse and mountain goat as early post-glacial colonizers.

South Coast Subregion –

Quadra Island - Quadra Island is east of Vancouver Island in the Inside Passage between Vancouver Island and mainland British Columbia. Modelling of identified early post-glacial marine terraces and subsequent archaeological excavations recovered artifacts associated with sediments dated to 10,740 ± 70 ^{14}C BP at the Crescent Road site and 10,940 ± 60 ^{14}C BP at Yeatman Bay [*35*, *131*, *132*]. Additionally, archaeological sites with early Holocene (ca. 11–10 ka calBP) components contained stemmed and leaf-shaped points and knives, and a unidirectional blade-like core reduction assemblage [*35*, *132*]. The Quadra Island case study demonstrated the effectiveness of post-glacial sea level modelling in archaeological investigations.

Strait of Georgia, British Columbia. Late Pleistocene aged Steller sea lion (*Eumetopias jubaus*) bones have been reported from the Strait of Georgia area. These include the radius of an immature Steller sea lion found in sewer excavations in Vancouver that is probably from late Pleistocene raised delta and marine deposits and specimens found at Bowen Island [*100*]. At Qualicum Beach, the partial skeleton of a juvenile Steller sea lion from Late Wisconsin Capilano

sediments had exhibited an oval puncture on the left parietal bone interpreted as a fatal wound inflicted by an adult Steller sea lion. The specimen was radiocarbon dated to 12,570 ± 70 ^{14}C BP [*100*]. These occurrences of Stellar sea lions likely indicate the presence of one or more rookeries [*100*].

Glenrose Cannery (DgRr-6). The Glenrose Cannery site is located on the banks of the Fraser River in Delta, British Columbia. This multicomponent shell midden site has been key in archaeological interpretations of the development of social complexity on the Northwest Coast [*133*, *134*]. The earliest archaeological component at the site dates to 8,150 ± 250 ^{14}C BP and includes leaf-shaped and stemmed bifaces, cobble tools, and a bone and antler tool assemblage. The faunal remains assigned to the early component include elk, deer, seal, and marine fish including salmon, and are indicative of a mixed terrestrial and marine diet [*134*].

Stave Lake. The Stave Watershed is situated inland on the upper Fraser delta in southwest British Columbia. A hydroelectric facility has inundated the former lake shoreline where there are numerous archaeological sites. Archaeological surveys during periodic draw-down have recovered a nearly continuous sequence of archaeological sites spanning back to the latest Pleistocene [*135*, *103*]. Six sites (DhRn-16, DhRn-18, DhRn-21, DhRn-29, DhRo-16, and DhRo-11) have radiocarbon dates or are assigned typologically to the Protowestern Tradition that dates from 11,340 ^{14}C BP to 8,500 ^{14}C BP [*103*]. At the Cardinalis Creek Mouth Site (DhRn-29), a chert biface preform and multiple flakes were found with charcoal collected directly under the biface that dated to 10,150 ± 40 ^{14}C BP [*103*]. Paleoindian projectile point types have found in the Stave Watershed and include one Clovis-style fluted point base and an Alberta point

fragment. The Clovis-style point base was found on the deflated surface of archaeological site DhRn-20 where no integral subsurface deposits have been identified and from which there are no radiocarbon dates [*103*].

Extinct megafauna, Southern Vancouver Island and Puget Sound. Fossil remains of extinct mammals, including Columbian mammoth (*Mammuthus columbi*, including *M. imperator*), American mastodon (*Mammut americanum*), and helmeted muskox (*Bootherium bombifrons*), have been found in sediments deposited before the glacial maximum on southeastern Vancouver Island and south to Puget Sound [*136*, *137*]. A giant short-faced bear partial ulna collected from the base of a Quaternary sediment exposure at Cowichan Head on the Saanich Peninsula was radiocarbon dated to 22,750 ± 140 ^{14}C BP [*99*]. The specimen had probably eroded from Quadra Sands that formed in massive aggregations as distal and marginal outwash aprons and in meltwater channels during the advance of the CIS. A partial mammoth humerus from a gravel pit near Cowichan Head dated to 17,000 ± 240 ^{14}C BP [*138*] is the latest dated vertebrate prior to full glacial ice cover during the glacial maximum.

The early post-glacial fauna from this area includes a short-faced bear partial mandible found in wetland deposits on San Juan Island in Washington [*101*], and at least twelve bison specimens from southern Vancouver Island and the San Juan Islands [*82*], including a specimen found in North Saanich directly dated to 11,750 ± 100 ^{14}C BP [*139*]. There are at least nine bison documented from six localities on Orcas Island. Three AMS radiocarbon dates on purified collagen from Orcas Island bison bones range from 11,760 ^{14}C BP to 10,800 ^{14}C BP [*101*]. The oldest bison specimens from Ayer Pond show evidence of human involvement (as is noted below) and were redated with updated AMS protocols resulting in an age of 11,990 ± 25 ^{14}C BP

[*70*]. This age signals an early post-glacial land mammal dispersal from the mainland to the San Juan Islands and southern Vancouver Island [*101*].

Cedar Hollow, Whidbey Island, Washington. Cedar Hollow is a paleontological site located on westernmost Whidbey Island. Early Holocene fossiliferous strata consist of dune sands that partially infill a kettle exposed in a coastal bluff. Two radiocarbon dates from this site are 8,280 ± 40 ^{14}C BP on a vole bone and 8,840 ± 50 ^{14}C BP on a deer bone [*140*]. The faunal assemblage includes deer, brown bear, grey wolf, deer mouse, Microtus voles, and six avian species.

Ayer Archaeological Site, Orcas Island (45SJ454). The fossil bison assemblage from the Ayer Site on Orcas Island includes two partial adult males, an adult female, and a juvenile found in early post-glacial lacustrine sediments below a peat layer and above Everson Glaciomarine Drift [*101*]. Sixty-one marks consistent with human-made modifications were identified on 34 of the Ayer bison bone specimens [*70*] and resulted in the site's archaeological designation. The modifications include two V-section groove chop marks, spirally fractured limb bones, and concentric sub-angular fractures around impact notches [*70*]. A radiocarbon date on a bison medial phalanx of 11,990 ± 25 ^{14}C BP provides an age for the modified specimens.

Manis Mastodon Site (45CA218). The Manis Mastodon archaeological site is located on the Olympic Peninsula in Clallam County near Sequim, Washington. A male mastodon (*Mammut americanum*) was excavated from sediments at the base of a glacial kettle pond [*68, 69*] and dates to 11,960 ± 17 ^{14}C BP [*69*]. Caribou, bison, and muskrat remains were also recovered at Manis [*141*]. Archaeological materials include several bones with flake removals or cut marks

suggestive of butchery by humans, a spall tool found with the mastodon remains, and a bone point embedded in one of the mastodon's ribs has been considered direct evidence that the animal was hunted [*68, 69*; although see *12*, *28*, *142*].

References

1. Haynes Jr., C. V. Fluted projectile points: Their age and dispersion. *Science* **145**, 1408-1413 (1966).

2. Haynes Jr., C. V. The earliest Americans. *Science* **166**, 709-715 (1969).

3. Martin, P. S. The discovery of America: The first Americans may have swept the Western Hemisphere and decimated its fauna within 1000 years. *Science* **179**, 969-974 (1973).

4. Fladmark, K. R. Routes: Alternate migration corridors for early man in North America. *Am. Antiq.* **44,** 55-69 (1979).

5. Mead, J. I. Is it really that old? A comment about the Meadowcroft Rockshelter "overview". *Am. Antiq.* **45**, 579-582 (1980).

6. Grayson, D. K. Archaeological associations with extinct Pleistocene mammals in North America. *J. Archaeol. Sci.* **11**, 213-221 (1984).

7. Dillehay, T. D. Monte Verde. *Science* 245, 1436 (1989).

8. Holliday, V. T. *Paleoindian Geoarchaeology of the Southern High Plains* (University of Texas Press, Austin, TX, 1997).

9. Meltzer, D. J. *et al*. On the Pleistocene antiquity of Monte Verde, southern Chile. *Am. Antiq.* **62**, 659-663 (1997).

10. Adovasio, J. M., Pedler, D. R., Donahue, J. & Stuckenrath, R. Two decades of debate on Meadowcroft Rockshelter. *North American Archaeologist* **19**, 317-341 (1999).

11. Dixon, E. J. *Bones, Boats, and Bison: Archeology and the First Colonization of Western North America* (University of New Mexico Press, Albuquerque, NM, 1999).

12. Grayson, D. K. & Meltzer, D. J. A requiem for North American overkill. *J. Archaeol. Sci.* **30**, 585-593 (2003).

13. Fiedel, S. J. & Haynes, G. A premature burial: Comments on Grayson and Meltzer's “Requiem for overkill”. *J. Archaeol. Sci.* **31**, 121-131 (2004).

14. Madsen, D. B. (ed.). *Entering America: Northeast Asia and Beringia before the Last Glacial Maximum* (The University of Utah Press, Salt Lake City, UT, 2004).

15. Erlandson, J. M. *et al*. The kelp highway hypothesis: Marine ecology, the coastal migration theory, and the peopling of the Americas. *J. Isl. Coastal Archaeol.* **2**, 161-174 (2007).

16. Firestone, R. B. *et al*. Evidence for an extraterrestrial impact 12,900 years ago that contributed to the megafaunal extinctions and the Younger Dryas cooling. *Proc. Natl. Acad. Sci. U.S.A.* **104**, 16016-16021 (2007).

17. Waters, M. R. & Stafford Jr., T. W. Redefining the age of Clovis: Implications for the peopling of the Americas. *Science* **315**, 1122-1126 (2007).

18. Tamm, E. *et al*. T Beringian standstill and spread of Native American founders. *PLOS ONE* **2**, e829 10.1371/journal.pone.0000829 (2007).

19. Haynes, G. *et al*. Comment on "Redefining the age of Clovis: Implications for the peopling of the Americas”. *Science* **317**, 320 (2007).

20. Dillehay, T. D. *et al*. Monte Verde: Seaweed, food, medicine, and the peopling of South America. *Science* **320**, 784-786 (2008).

21. Goebel, T., Waters, M. R. & O’Rourke, D. H. The late Pleistocene dispersal of modern humans in the Americas. *Science* **319**, 1497 (2008).

22. Meltzer, D. J. *First Peoples in a New World: Colonizing Ice Age America* (University of California, Berkeley, CA, 2009).

23. Jenkins, D. L. Clovis age Western Stemmed projectile points and human coprolites at the Paisley Caves. *Science* **337**, 223-228 (2012).

24. Stanford, D. J. & Bradley, B. *Across Atlantic Ice: The Origin of America's Clovis Culture* (University of California Press, Berkeley, CA, 2012).

25. Graf, K. E., Ketron, C. V. & Waters, M. R. (eds.). *Paleoamerican Odyssey* (Texas A&M Univ. Press, College Station, TX, 2014).

26. Holliday, V. T., Meltzer, D. J., Grayson, D. K., Surovell, T. & Boslough, M. The Younger Dryas impact hypothesis: A cosmic catastrophe. *J. Quat. Sci.* **29**, 525-530 (2014).

27. O'Brien, M. *et al*. On thin ice: Problems with Stanford and Bradley's proposed Solutrean colonisation of North America. *Antiquity* **88**, 606-613 (2014).

28. Grayson, D. K. & Meltzer, D. J. Revisiting Paleoindian exploitation of extinct North American mammals. *J. Archaeol. Sci.* **56**, 177-193 (2015).

29. Hoffecker, J. F., Elias, S. A., O'Rourke, D. H., Scott, G. R. & Bigelow, N. H. Beringia and the global dispersal of modern humans. *Evol. Anthropol.* **25**, 64-78, 2016.

30. Braje, T. J., Dillehay, T.D., Erlandson, J. M., Klein, R. G. & Rick, T. C. Finding the first Americans. *Science* **358**, 592–594 (2017).

31. Bourgeon, L. Burke, A. & Higham, T. Earliest human presence in North America dated to the Last Glacial Maximum: New radiocarbon dates from Bluefish Caves, Canada. *PLOS ONE* **12**, e0169486 10.1371/journal.pone.0169486 (2017).

32. Waters, M. R. *et al*. Pre-Clovis projectile points at the Debra L. Friedkin site, Texas—Implications for the late Pleistocene peopling of the Americas. *Sci. Adv.* **4**, eaat4505 10.1126/sciadv.aat4505 (2018).

33. Moreno-Mayar, J. V. *et al*. Early human dispersals within the Americas. *Science* **362**, 1-28 (2018).

34. Potter, B. A. *et al*. Current evidence allows multiple models for the peopling of the Americas. *Sci. Adv.* **4**, eaat5473 10.1126/sciadv.aat5473 (2018).

35. McLaren, D. *et al*. Late Pleistocene archaeological discovery models on the Pacific Coast of North America. *PaleoAmerica* **6**, 43-63 (2019).

36. Waters, M. R. Late Pleistocene exploration and settlement of the Americas by modern humans. *Science* **365**, eaat5447 10.1126/science.aat5447 (2019).

37. Davis, L. G. & Madsen, D. B. The coastal migration theory: Formulation and testable hypotheses. *Quat. Sci. Rev.* **249**, 106605 10.1016/j.quascirev.2020.106605 (2020).

38. Davis, L. G. *et al*. Dating of a large tool assemblage at the Cooper's Ferry site (Idaho, USA) to ~15,785 cal yr B.P. extends the age of stemmed points in the Americas. *Sci. Adv.* **8**, eade1248 10.1126/sciadv.ade1248 (2022).

39. Dillehay. T. D. Monte Verde: A late Pleistocene settlement in Chile, the archaeological context and interpretation, vol. 2 (Smithsonian Institution Press, Washington, DC, 1997).

40. Waters, M. R. *et al*. The Buttermilk Creek Complex and the origins of Clovis at the Debra L. Friedkin site, Texas. *Science* **331**, 1599-1603 (2011).

41. Halligan, J. J. *et al.* Pre-Clovis occupation 14,550 years ago at the Page-Ladson site, Florida, and the peopling of the Americas. *Sci. Adv.* **2**(5), e1600375 10.1126/sciadv.1600375 (2016).

42. Dillehay, T. D. *et al.* Simple technologies and diverse food strategies of the late Pleistocene and early Holocene at Huaca Prieta, coastal Peru. *Sci. Adv.* **3**, e1602778 10.1126/sciadv.160277 (2017).

43. Williams, T. J. *et al*. Evidence of an early projectile point technology in North America at the Gault Site, Texas, USA. *Sci. Adv.* **4**(7), 10.1126/sciadv.aar5954 (2018).

44. Davis, L. G. *et al*. Late Upper Paleolithic occupation at Cooper's Ferry, Idaho, USA, ~16,000 years ago. *Science* **365**, 891-897 (2019).

45. Clark, P. U. *et al*. The Last Glacial Maximum. *Science* **325**, 710-714 (2009).

46. Clark, J. *et al*. The age of the opening of the Ice-Free Corridor and implications for the peopling of the Americas. *Proc. Natl. Acad. Sci. U.S.A.* **119**, e2118558119 10.1073/pnas.211855811 (2022).

47. Dyke, A. S., Moore, A. & Robertson, L. *Deglaciation of North America. Geol. Surv. Can. Open File 1574* (2003).

48. Dalton, A. S. *et al*. An updated radiocarbon-based ice margin chronology for the last deglaciation of the North American Ice Sheet Complex. *Quat. Sci. Rev.* **234**, 106223 10.1016/j.quascirev.2020.106223 (2020).

49. Burns, J. A. Mammalian faunal dynamics in late Pleistocene Alberta, Canada. *Quat. Int.* **217**, 37-42 (2010).

50. Heintzman, P. D. *et al*. Bison phylogeography constrains dispersal and viability of the Ice Free Corridor in western Canada. *Proc. Natl. Acad. Sci. U.S.A.* **113**, 8057-8063 (2016).

51. Pedersen, M. W. *et al*. Postglacial viability and colonization in North America's ice-free corridor. *Nature* **537**, 45-49 (2016).

52. Mandryk, C., Josenhans, H., Fedje, D. & Mathewes, R. Late Quaternary paleoenvironments of Northwestern North America: Implications for inland versus coastal migration routes. *Quat. Sci. Rev.* **20**, 301-314 (2001).

53. Mackie, Q., Fedje, D., & McLaren, D. Archaeology and sea level change on the British Columbia Coast. *CJA* **42**, 74-91 (2018).

54. Bennett, M. R. *et al*. Evidence of humans in North America during the Last Glacial Maximum. *Science* **373**, 1528–1531 (2021).

55. Madsen, D. B., Davis, L. G., Rhode, D. & Oviatt, C. G. Comment on "Evidence of humans in North America during the Last Glacial Maximum". *Science* **374**, eabm4678 10.1126/science.abg7586 (2022).

56. Haynes Jr., C. V. Evidence for humans at White Sands National Park during the Last Glacial Maximum could actually be for Clovis people ~13,000 years ago. *PaleoAmerica* **8**, 95-98 (2022).

57. Pigati, J. S. *et al*. Response to Comment on "Evidence of humans in North America during the Last Glacial Maximum". *Science* **375**, eabm6987 10.1126/science.abm6987 (2022).

58. Pigati, J. S. *et al*. Reply to "Evidence for humans at White Sands National Park during the Last Glacial Maximum could actually be for Clovis people ~13,000 Years Ago" by C. Vance Haynes, Jr. *PaleoAmerica* **8**, 99-101 (2022).

59. Rachal, D. M., Mead, J. I., Dello-Russo, R. & Cuba, M. T. Deep-water delivery model of *Ruppia* seeds to a nearshore/terrestrial setting and its chronological implications for late Pleistocene footprints, Tularosa Basin, New Mexico. *Geoarchaeology* **37**, 923-933 (2022).

60. Oviatt, C., Madsen, D., Rhode, D. & Davis, L. A. critical assessment of claims that human footprints in the Lake Otero basin, New Mexico date to the Last Glacial Maximum. *Quat. Res.* **111**, 138-147 (2023).

61. Rachal, D. M., Dello-Russo, R. & Cuba, M. The Pleistocene footprints are younger than we thought: Correcting the radiocarbon dates of *Ruppia* seeds, Tularosa Basin, New Mexico. *Quat. Res*. 1-12 (2024).

62. Pigati, J. S. *et al*. Independent age estimates resolve the controversy of ancient human footprints at White Sands. *Science* **382**, 73-75 (2023).

63. Clague, J. J. & Ward, B. Pleistocene Glaciation of British Columbia. *Dev. Quat. Sci.* **15**, 563-573 (2011).

64. Bronk Ramsey, C. Bayesian analysis of radiocarbon dates. *Radiocarbon* **51**, 337-360 (2009).

65. Reimer, P. *et al*. The IntCal20 Northern Hemisphere radiocarbon age calibration curve (0–55 cal kBP). *Radiocarbon* **62** (2020).

66. Heaton, T. J. *et al*. Marine20—the marine radiocarbon age calibration curve (0–55,000 calBP). *Radiocarbon* **62**, 779-820 (2020).

67. Ward, B. C. *et al*. Port Eliza cave: North American west coast interstadial environment and implications for human migrations. *Quat. Sci. Rev.* **22**, 1383-1388 (2003).

68. Gustafson, C. E. Gilbow, D. & Daugherty, R. The Manis Mastodon Site: Early man on the Olympic Peninsula. *CJA* **3**, 157-164 (1979).

69. Waters, M. R. et al. Pre-Clovis mastodon hunting 13,800 years ago at the Manis Site, Washington. *Science* **334**, 351-353 (2011).

70. Kenady, S. M., Wilson, M. C., Schalk, R. F. & Mierendorf, R. R. Late Pleistocene butchered *Bison antiquus* from Ayer Pond, Orcas Island, Pacific Northwest: Age confirmation and taphonomy. Quat. Int., 233, 130-141 (2011).

71. Clague, J. J. & James, T. S. History and isostatic effects of the last ice sheet in southern British Columbia. *Quat. Sci. Rev.* **21**, 71-87 (2002).

72. Shugar, D. H. *et al*. Post-glacial sea-level change along the Pacific coast of North America. *Quat. Sci. Rev.* **97**, 170-192 (2014).

73. Mathewes, R. W. & Clague, J. J. Paleoecology and ice limits of the early Fraser Glaciation (Marine Isotope Stage 2) on Haida Gwaii, British Columbia, Canada. *Quat. Res.* **88**, 277-292 (2017).

74. Clague, J. J., Mathewes, R.W. & Agar, T. A. Environments of Northwestern North America before the Last Glacial Maximum in *Entering America: Northeast Asia and Beringia before the Last Glacial Maximum* (The University of Utah Press, Salt Lake City, UT, 2004), pp. 63-94.

75. Mann, D. H. Wisconsin and Holocene glaciation of Southeast Alaska in *Glaciation in Alaska* (Alaska Geological Society, Anchorage, AK 1986), pp. 237-265.

76. Carrara, P. E., Ager, T. A. & Baichtal, J. F. Possible refugia in the Alexander Archipelago of southeastern Alaska during the Late Wisconsin glaciation. *Can. J. Earth Sci.* **44**, 229-244 (2007).

77. Heaton, T. H. & Grady, F. The Late Wisconsin vertebrate history of Prince of Wales Island, Southeast Alaska in *Ice Age Cave Faunas of North America* (Indiana University Press, Bloomington, IN, 2003), pp. 17-53.

78. Dixon, E. J. *et al*. Late Quaternary regional geoarchaeology of Southeast Alaska karst: A progress report. *Geoarchaeology* **12**, 689-712 (1997).

79. Lesnek, A. J., Briner, J.P., Lindqvist, C., Baichtal, J. F. & Heaton, T. H. Deglaciation of the Pacific coastal corridor directly preceded the human colonization of the Americas. *Sci. Adv.* **4**, eaar5040 10.1126/sciadv.aar5040 (2018).

80. Blaise, B. Clague, J. J. & Mathewes, R.W. Time of maximum Late Wisconsinan Glaciation, west coast of Canada. *Quat. Res.* **47**, 140-146 (1990).

81. Ramsey, C., Griffiths. P., Fedje, D., Wigen, R. & Mackie, Q. Preliminary investigation of a Late Wisconsinan fauna from K1 Cave, Queen Charlotte Islands (Haida Gwaii), Canada. *Quat. Res.* **62**, 105-109 (2004).

82. Mackie, Q., Fedje, D., McLaren, D., Smith, N., McKechnie, I. Early Environments and Archaeology of Coastal British Columbia in *Trekking the Shore:Changing Coastlines and the Antiquity of Coastal Settlement* (Springer, New York, NY, 2011), pp. 51-103.

83. Fedje, D. W., Mackie, Q., Lacourse, T. & McLaren, D. Younger Dryas environments and archaeology on the Northwest Coast of North America. *Quat. Int.* **242**, 452-462 (2011).

84. Fedje, D., Mackie, Q., Smith, N. & McLaren, D. Function, visibility, and interpretation of archaeological assemblages at the Pleistocene/Holocene transition in Haida Gwaii in *From the Yenisei to the Yukon: Interpreting Lithic Assemblage Variability in Late Pleistocene/Early Holocene Beringia* (Texas A&M University Press, College Station, TX, 2011), pp. 323-342.

85. Cosma, T. N., Hendy, I. L. & Chang, A. S. Chronological constraints on Cordilleran Ice Sheet glaciomarine sedimentation from core MD02-2496 off Vancouver Island (Western Canada). *Quat. Sci. Rev.* **27**, 941-955 (2008).

86. Darvill, C. M., Menounos, B., Goehring, B. M., Lian, O. B. & Caffee M. W. Retreat of the western Cordilleran Ice Sheet margin during the last deglaciation. *Geophys. Res. Lett.* **45**, 9710-9720 (2018).

87. Howes, D. E. Late Quaternary sediments and geomorphic history of northern Vancouver Island, British Columbia. *Can. J. Earth Sci.* **20**, 57-65 (1983).

88. Hebda, R. J. Late-glacial and postglacial vegetation history at Bear Cove bog, Northeast Vancouver Island, British Columbia. *Can. J. Bot.* **61**, 3172-3192 (1983).

89. Mathews, W. H., Fyles, J. G. & Nasmith, H. W. Postglacial crustal movements in southwestern British Columbia and adjacent Washington State. *Can. J. Earth Sci.* **7**, 690-702 (1970).

90. Steffen, M. L. & Fulton, T. L. On the association of giant short-faced bear (*Arctodus simus*) and brown bear (*Ursus arctos*) in late Pleistocene North America. *Geobios* **51**, 61-74 (2018).

91. Steffen, M. L. Late Pleistocene heather vole, *Phenacomys*, on the North Pacific Coast of North America: Environments, local extinctions, and archaeological implications. *Can. J. Earth Sci.* **59**, 708-721 (2022).

92. Al-Suwaidi, M. et al. Late Wisconsinan Port Eliza Cave sediments and their implications for human coastal migration, Vancouver Island, Canada. *Geoarcheology* 21, 307-332 (2006).

93. Shafer, A. B., Côté S. D. & Coltman, D. W. Hot spots of genetic diversity descended from multiple Pleistocene refugia in an alpine ungulate. *Evolution* **65**, 125-38 (2011).

94. Sawyer, Y. E., Flamme, M. J., Jung, T. S., MacDonald S. O. & Cook J. A. Diversification of deer mice (Rodentia: genus *Peromyscus*) at their north-western range limit: Genetic consequences of refugial and island isolation. *J. Biogeogr.* **44**, 1572-1585 (2017).

95. Luternauer, J. L. & Murray, J. W. *Late Quaternary morphologic development and sedimentation, central British Columbia continental shelf* (Paper 83-21, GSC, 1983).

96. Herzer, R. H. & Bornhold, B. D. Glaciation and Post-Glacial History of the Continental Shelf off southwestern Vancouver Island, British Columbia. *Mar. Geol.* **48**, 285-319 (1982).

97. Luternauer, J. L. *et al*. Late Pleistocene terrestrial deposits on the continental shelf of western Canada: Evidence for rapid sea-level change at the end of the last glaciation. *Geology* **17**, 357-360 (1989).

98. Porter, S. C. & Swanson, T. W. Radiocarbon age constraints on rates of advance and retreat of the Puget lobe of the Cordilleran Ice Sheet during the last glaciation. *Quat. Res.* **50**, 205-213 (1998).

99. Steffen, M. L. & Harington, C. R. Giant short-faced bear (*Arctodus simus*) from Late Wisconsinan deposits at Cowichan Head, Vancouver Island, British Columbia. *Can. J. Earth Sci.* **47**, 1029-1036 (2010).

100. Harington, C. R. Ross, R. L. M. Mathewes, R. W. Stewart, K. M. & Beattie, O. 2004. A late Pleistocene Steller sea lion (*Eumetopias jubatus*) from Courtenay, British Columbia: Its death, associated biota, and paleoenvironment. *Can. J. Earth Sci.* **41**, 1285-1297.

101. Wilson, M. C., Kenady, S. M. & Schalk, R. F. Late Pleistocene *Bison antiquus* from Orcas Island, Washington, and the biogeographic importance of an early postglacial land mammal dispersal corridor from the mainland to Vancouver Island. *Quat. Res.* 71, 49-61 (2009).

102. Croes, D. R. *et al.* The Projectile Point Sequences in the Puget Sound Region in *Projectile Point Sequences in Northwestern North America* (Archaeology Press, Simon Fraser University, Burnaby, BC, 2008), pp. 105–130.

103. McLaren, D. The occupational history of the Stave Watershed in *Archaeology of the Lower Fraser River Region* (Archaeology Press, Simon Fraser University, Burnaby, BC, 2017), pp. 149-158.

104. Gramly, R. M. *The Richey Clovis Cache*: *Earliest Americans Along the Columbia River* (Persimmon Press, Buffalo, NY, 1993).

105. Kilby, J. D. & Huckell, B. B. Clovis caches: Current perspectives and future directions in *PaleoAmerican Odyssey* (Texas A&M University Press, Austin, TX, 2014), pp. 257-272.

106. Reimer, P. J. & Reimer, R. W. A marine reservoir correction database and on-line interface. *Radiocarbon* **43**, 461-463 (2001).

107. Heaton, T. *et al*. A response to community questions on the Marine20 radiocarbon age calibration curve: Marine reservoir ages and the calibration of ^{14}C samples from the oceans. *Radiocarbon* **65**, 247-273 (2023).

108. Edge, D. *et al*. A modern multicentennial record of radiocarbon variability from an exactly dated bivalve chronology at the Tree Nob site (Alaska). *Radiocarbon* **65**, 81-96 (2022).

109. Fernandes, R., Millard, A. R., Brabec, M., Nadeau, M.-J. & Grootes, P. Food reconstruction using isotopic transferred signals (FRUITS): A Bayesian model for diet reconstruction. *PLOS ONE* **9**, e87436 10.1371/journal.pone.0087436 (2014).

110. Keeling, R. F. *et al*. Atmospheric evidence for a global secular increase in carbon isotopic discrimination of land photosynthesis. *Proc. Natl. Acad. Sci. U.S.A.* **114**, 10361-10366 (2017).

111. Bocherens, H. Isotopic tracking of large carnivore palaeoecology in the mammoth steppe. *Quat. Sci. Rev.* **117**, 42-71 (2015).

112. Dixon, E. J. Human colonization of the Americas: Timing, technology and process. *Quat. Sci. Rev.* **20**, 277-299 (2001).

113. Davis, S. D. (ed.). *The Hidden Falls Site, Baranof Island, Alaska* (Alaska Anthropological Association Monograph Series, vol. 5, Brockport, 1989).

114. Ackerman, R. E., Ground Hog Bay, site 2 in *American Beginnings: The Prehistory and Palaeoecology of Beringia* (University of Chicago Press, Chicago, IL, 1996), pp. 424-429.

115. Ackerman, R. E., Hamilton, T. D. & Stuckenrath, R. Early culture complexes on the Northern Northwest Coast. *CJA* **3**, 195-209 (1979).

116. Mathewes, R. W., Richards, M. & Reimchen, T. E. Late Pleistocene age, size, and paleoenvironment of a caribou antler from Haida Gwaii, British Columbia. *Can. J. Earth Sci.* **56**, 688-692 (2019).

117. Mathewes, R. W., Lian, O. Clague, J. J. & Huntley, M. Early Wisconsinan (MIS 4) Glaciation on Haida Gwaii, British Columbia, and implications for biological refugia. *Can. J. Earth Sci.* **52**, 939-951 (2015).

118. Fedje, D.W., Mackie, Q., McLaren, D. S. & Christensen, T. A. projectile point sequence for Haida Gwaii in *Projectile Point Sequences in Northwestern North America* (Archaeology Press, Simon Fraser University, Burnaby, BC, 2008), pp. 19-40.

119. Steffen, M. L. & Mackie, Q. An experimental approach to understanding burnt fish bone assemblages within archaeological hearth contexts. *Can. Zooarch.* **23**, 11-38 (2005).

120. Mackie, A. P. & Sumpter, I. D. Shoreline settlement patterns in Gwaii Haanas during the early and late Holocene in *Haida Gwaii, Human History and Environment from the Time of Loon to the Time of the Iron People* (UBC Press, Vancouver, BC, 2005), pp. 337-371.

121. Carlson, R. L. Early Namu in *Early Human Occupation in British Columbia* (UBC Press, Vancouver, BC, 1995), pp. 83-102.

122. Cannon, A. *The Economic Prehistory of Namu: Patterns in Vertebrate Fauna* (Archaeology Press, Simon Fraser University, Burnaby, BC, 1991).

123. Cannon, A. Settlement and sea-levels on the Central Coast of British Columbia: Evidence from shell midden cores. *Am. Antiq.* **65**, 67-77 (2000).

124. McLaren, D. *et al*. Terminal Pleistocene Epoch human footprints from the Pacific Coast of Canada. *PLOS ONE* **13**, e0193522 10.1371/journal.pone.0193522 (2018).

125. Gauvreau, A. *et al*. Geo-archaeology and Haíłzaqv oral history: Long-term human investment and resource use at EkTb-9, Triquet Island, N̓úláw̓itxˇv tribal area, Central Coast, British Columbia, Canada. *J. Archaeol. Sci. Rep.* **49**, 103884 10.1016/j.jasrep.2023.103884 (2023).

126. Carlson, C. C. The Bear Cove fauna and the subsistence history of Northwest Coast maritime culture in *Archaeology of Coastal British Columbia: Essays in Honour of Professor Philip M. Hobler* (Simon Fraser University Archaeology Press, Burnaby, BC, 2003), pp. 65-86.

127. Carlson, R. L. Cultural antecedents in *Handbook of North American Indians, Vol. 7, Northwest Coast* (Smithsonian Institution Press, Washington, D.C., 1990), pp. 60-69.

128. Steffen, M. L. Body-size trends in *Peromyscus* (Rodentia: Cricetidae) on Vancouver Island, Canada, with comments on relictual gigantism. *Paleobiology* **42**, 532-546 (2016).

129.Nagorsen, D. W. & Keddie, G. Late Pleistocene mountain goats (*Oreamnos americanus*) from Vancouver Island: Biogeographic implications. *J. Mammal.* **81**, 666-675 (2000).

130. Nagorsen, D. W., Keddie, G. & Hebda, R. J. Early Holocene black bears, *Ursus americanus*, from Vancouver Island. *Can. Field-Nat.* **109**, 11-18 (1995).

131.Fedje, D. *et al*. A revised sea level history for the northern Strait of Georgia, British Columbia, Canada. *Quat. Sci. Rev.* **192**, 300-316 (2018).

132. Fedje, D. *et al*. Slowstands, stillstands and transgressions: Paleoshorelines and archaeology on Quadra Island, BC, Canada. *Quat. Sci. Rev.* **270**, 107161 10.1016/j.quascirev.2021.107161 (2021).

133.Matson, R. G. The Old Cordilleran component at the Glenrose Cannery site in *Early Human Occupation in British Columbia* (UBC Press, Vancouver, BC, 1996), pp. 111-122.

134. Matson, R. G. & Coupland, G. *The Prehistory of the Northwest Coast* (Academic Press, San Diego, CA, 1996).

135. McLaren, D. & Steffen, M. L. A sequence of formed bifaces from the Fraser Valley Region of British Columbia in *Projectile Point Sequences in Northwestern North America* (Archaeology Press, Simon Fraser University, Burnaby, BC, 2008), pp. 87-104.

136. Harington, C. R. Quaternary animals: Vertebrates of the ice age in *Life in Stone: A Natural History of British Columbia's Fossils* (UBC Press, Vancouver, BC, 1996), pp. 259-273.

137. Barton, B. R. Notes on the new Washington State fossil, *Mammuthus columbi*. *Wash. Geol.* **26**, 68 (1998).

138. Blake Jr., W. *Geological Survey of Canada radiocarbon dates XXII* (*Paper 82-7, Geol. Surv. Can.,* 1982).

139. Hebda, R. J. & Spalding, D. A. E. Fossils and museums: Windows into ancient worlds in *Life in Stone: A Natural History of British Columbia's Fossils* (UBC Press, Vancouver, BC, 1996), pp. 14-24.

140. Mustoe, G. E., Harington, C. R. & Morlan, R. E. Cedar Hollow, an early Holocene faunal site from Whidbey Island, Washington. *West. N. Am. Nat.* **65**, 429-440 (2005).

141. Petersen, K. L. Mehringer, P. J. & Gustafson, C. E. Late-glacial vegetation and climate at the Manis Mastadon Site, Olympic Peninsula, Washington. *Quat. Res.* **20**, 215-231 (1983).

142. Haynes Jr., C. V. & Huckell, B. B. The Manis mastodon: An alternative interpretation. *PaleoAmerica* **2**, 189-191 (2016).

143. Lehner, N. S. *Arctic fox winter movements and diet in relation to industrial development on Alaska's North Slope* (University of Alaska Fairbanks, AK, 2012).

144. West, C. F. & France, C. A. Human and canid dietary relationships: Comparative stable isotope analysis from the Kodiak Archipelago, Alaska. *J. Ethnobiol.* **35**, 519-535 (2015).

145. Plint, T., Longstaffe, F. J., Ballantyne, A., Telka, A. & Rybczynski, N. Evolution of woodcutting behaviour in Early Pliocene beaver driven by consumption of woody plants. *Sci. Rep.* **10**, 13111 10.1038/s41598-020-70164-1 (2020).

146. Steffen, M. L. Coast-proximal inland archaeology and the Vancouver Island marmot (*Marmota vancouverensis*). *J. Archaeol. Sci. Rep*. **36**, 102863 10.1016/j.jasrep.2021.102863 (2021).

147. Jürgensen, J. *et al*. Diet and habitat of the saiga antelope during the late Quaternary using stable carbon and nitrogen isotope ratios. *Quat. Sci. Rev.* **160**, 150-161 (2017).

148. Fox-Dobbs, K., Leonard, J. A. & Koch, P. L. Pleistocene megafauna from eastern Beringia: Paleoecological and paleoenvironmental interpretations of stable carbon and nitrogen isotope and radiocarbon records. *Palaeogeogr. Palaeoclimatol. Palaeoecol.* **261**, 30-46 (2008).

149. Byers D. A., Yesner, D. R., Broughton, J. M. & Coltrain, J. B. Stable isotope chemistry, population histories and late Prehistoric subsistence change in the Aleutian Islands. *J. Archaeol. Sci.* **38**, 183-196 (2011).

150. Coltrain, J. B., Hayes, M. G. & O'Rourke, D. H. Sealing, whaling and caribou: The skeletal isotope chemistry of eastern Arctic foragers. *J. Archaeol. Sci.* **31**, 39-57 (2004).

151. Moss, M. L. *et al*. Historical ecology and biogeography of North Pacific pinnipeds: Isotopes and ancient DNA from three archaeological assemblages. *J. Island Coast. Archaeol.* **1**, 165-190 (2006).

152. Szpak, P., Orchard, T. J., McKechnie, I. & Gröcke, D. R. Historical ecology of late Holocene sea otters (*Enhydra lutris*) from northern British Columbia: Isotopic and zooarchaeological perspectives. *J. Archaeol. Sci.* **39**, 1553-1571 (2011).

153. McNeely, R., Dyke, A. S. & Southon, J. R. *Canadian Marine Reservoir Ages, Preliminary Data Assessment. Geol. Surv. Can. Open File 5049* (2006).

154. Robinson, S. W. & Thompson, G. Radiocarbon corrections for marine shell dates with application to southern Pacific Northwest Coast prehistory. *Syesis* **14**, 45-57 (1981).

Supplementary figures S1 to S4

Figure S1.

Southeast Alaska chronology showing radiocarbon lab numbers. Bayesian model of 74 calibrated ^{14}C ages on vertebrates from 15 caves and on bone and charcoal from three archaeological sites in Alexander Archipelago. Each curve indicates a single sample distribution. Calibration and Bayesian modeling are based on OxCal v 4.4.4 [*64*]. The grey vertical band indicates the CIS ice maximum and unlikely human settlement.

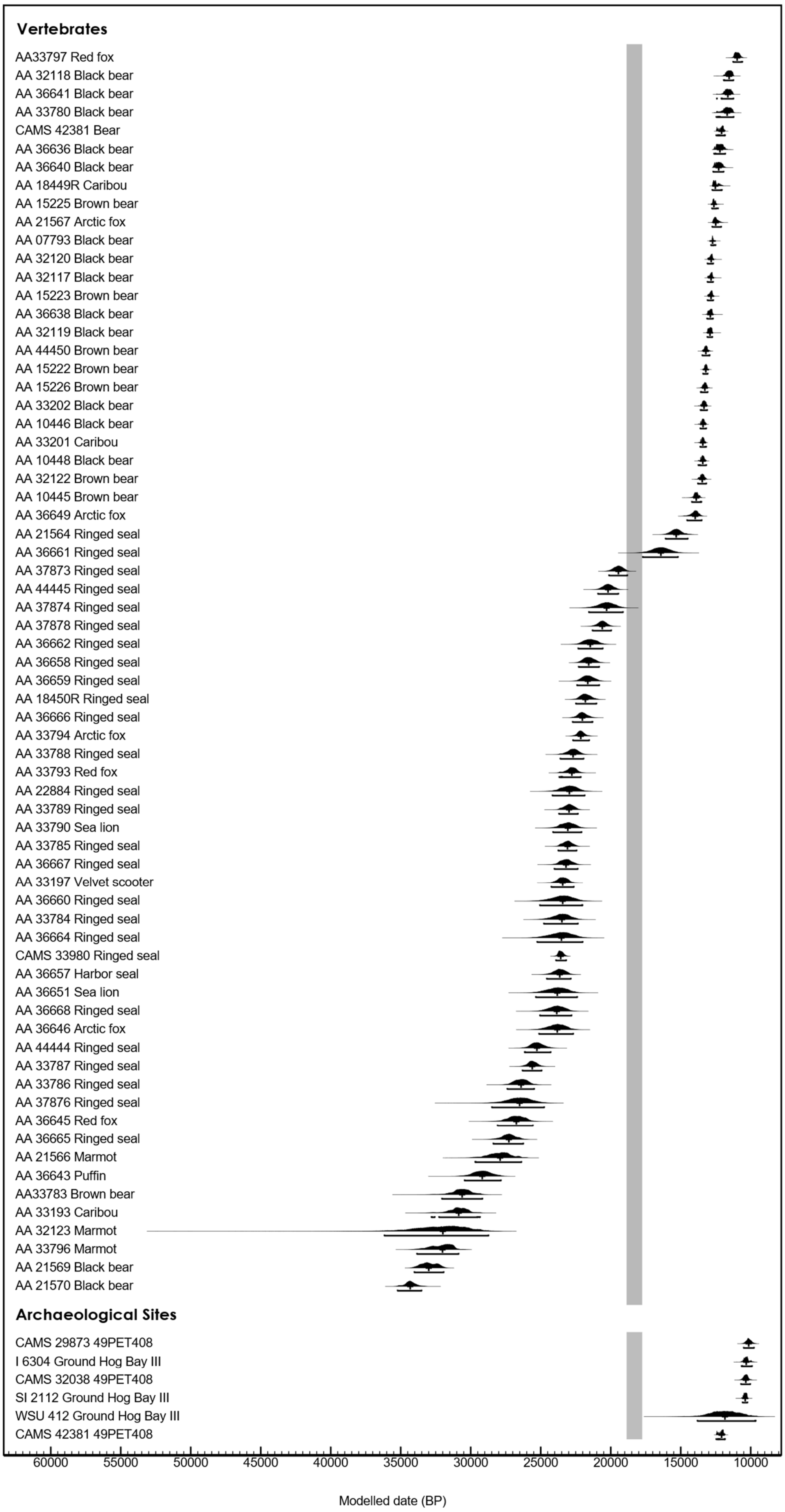

Supplementary figures S1 to S4

Figure S2.

Haida Gwaii chronology showing radiocarbon lab numbers. Bayesian model of 66 calibrated ^{14}C ages on vertebrates and on bone and charcoal from archaeological sites on Haida Gwaii. Each curve indicates a single sample distribution. Calibration and Bayesian modeling are based on OxCal v 4.4.4 [*64*]. The grey vertical band indicates the CIS ice maximum and unlikely human settlement.

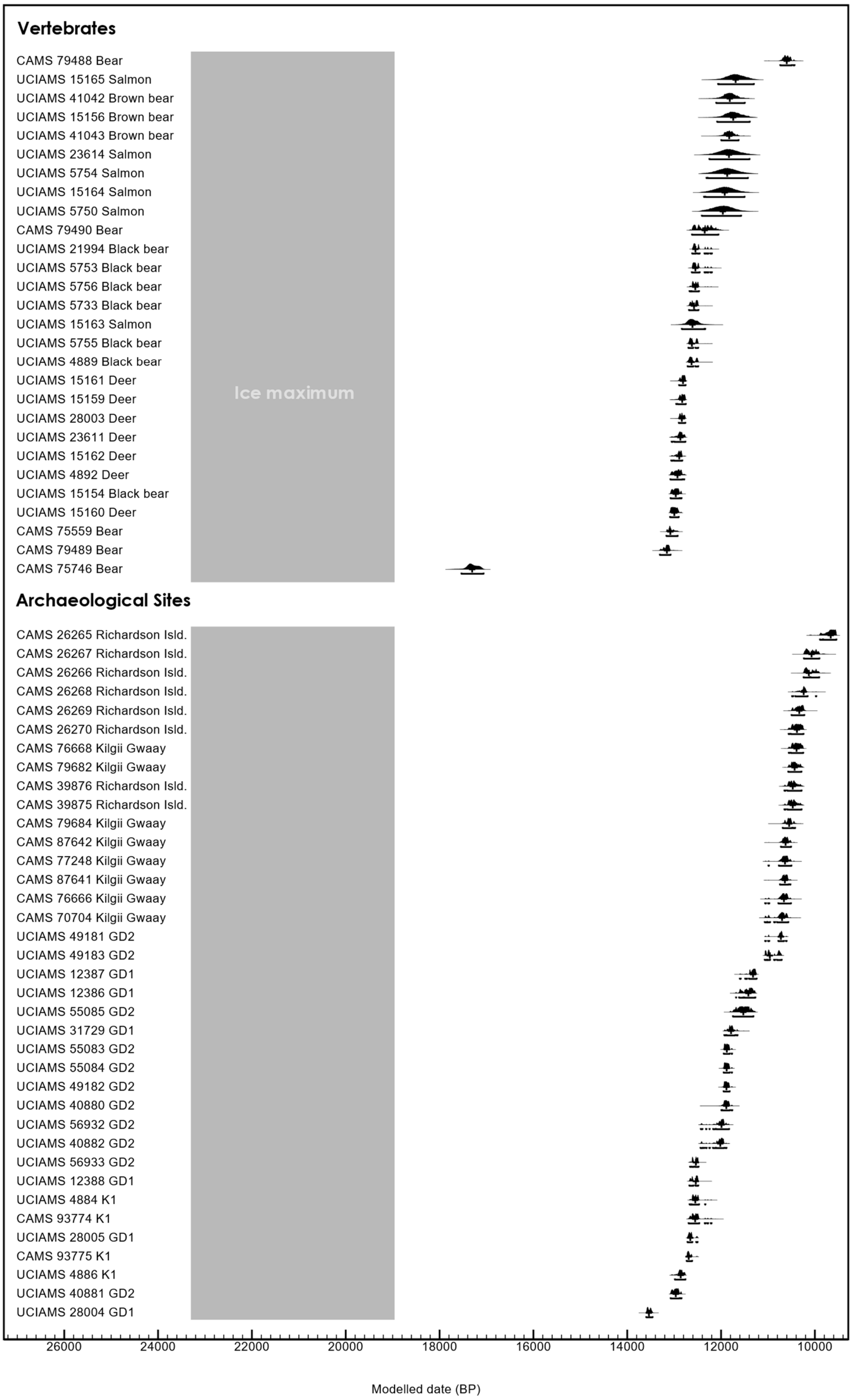

Supplementary figures S1 to S4

Figure S3.

Central coast chronology showing radiocarbon lab numbers. Bayesian model of 46 calibrated ^{14}C ages on vertebrates and on bone and charcoal from archaeological sites in the central coast subregion of the north Pacific coast. Each curve indicates a single sample distribution. Calibration and Bayesian modeling are based on OxCal v 4.4.4 [*64*]. The dark grey vertical band indicate the CIS maximum in the Queen Charlotte Sound and north Vancouver Island, and the light grey vertical band indicates ice maximum at north Vancouver Island (see manuscript text). Human settlement was impeded during the ice maximum.

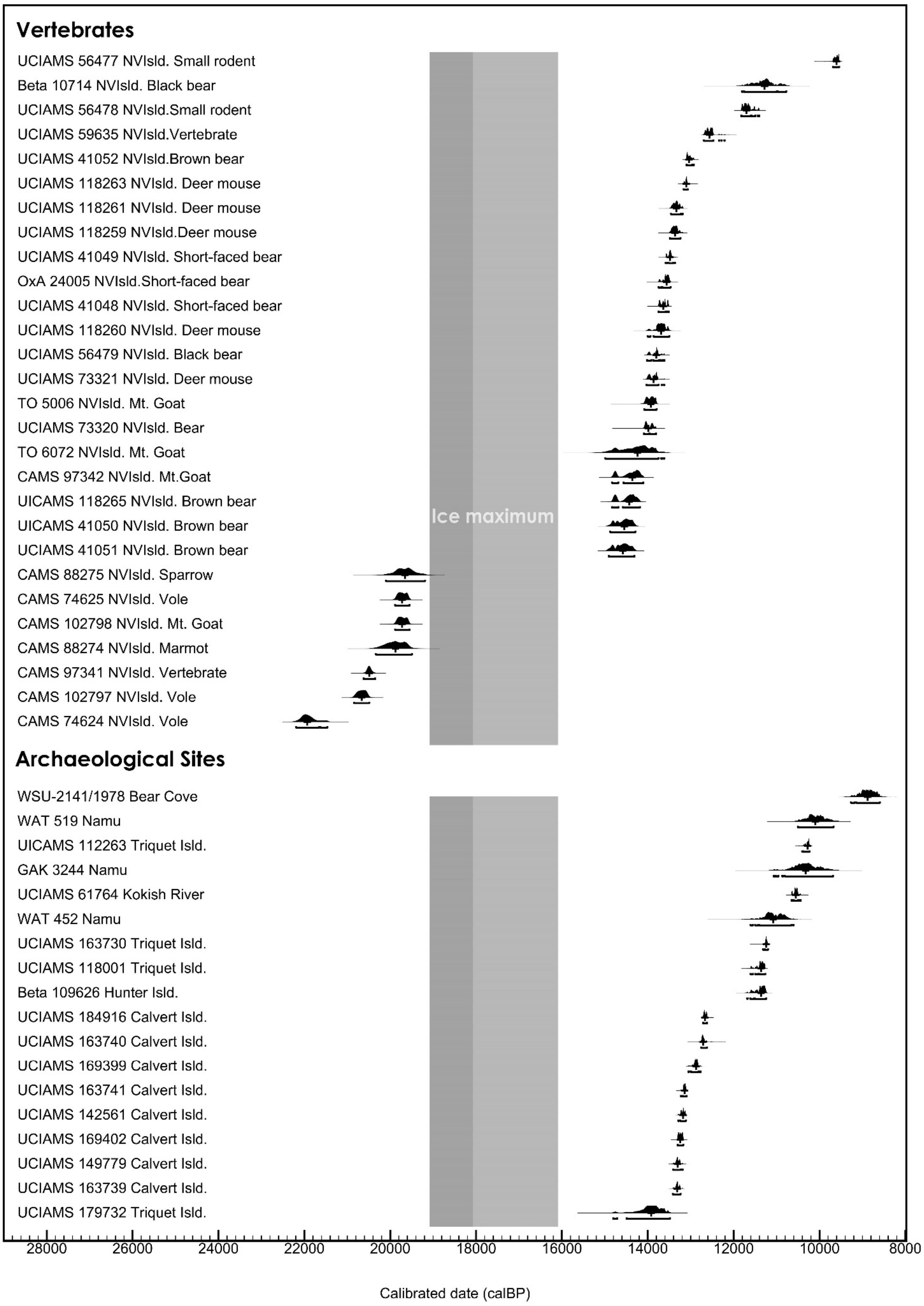

Supplementary figures S1 to S4

Figure S4.

South coast chronology showing radiocarbon lab numbers. Bayesian model of 47 calibrated ^{14}C ages on bones of vertebrates and on bone and charcoal from archaeological sites in the south coast subregion of the north Pacific coast. Each curve indicates a single sample distribution. Clovis start and end are from [*17*]. Calibration and Bayesian modeling are based on OxCal v 4.4.4 [*64*]. The grey vertical band indicates the CIS ice maximum and unlikely human settlement.

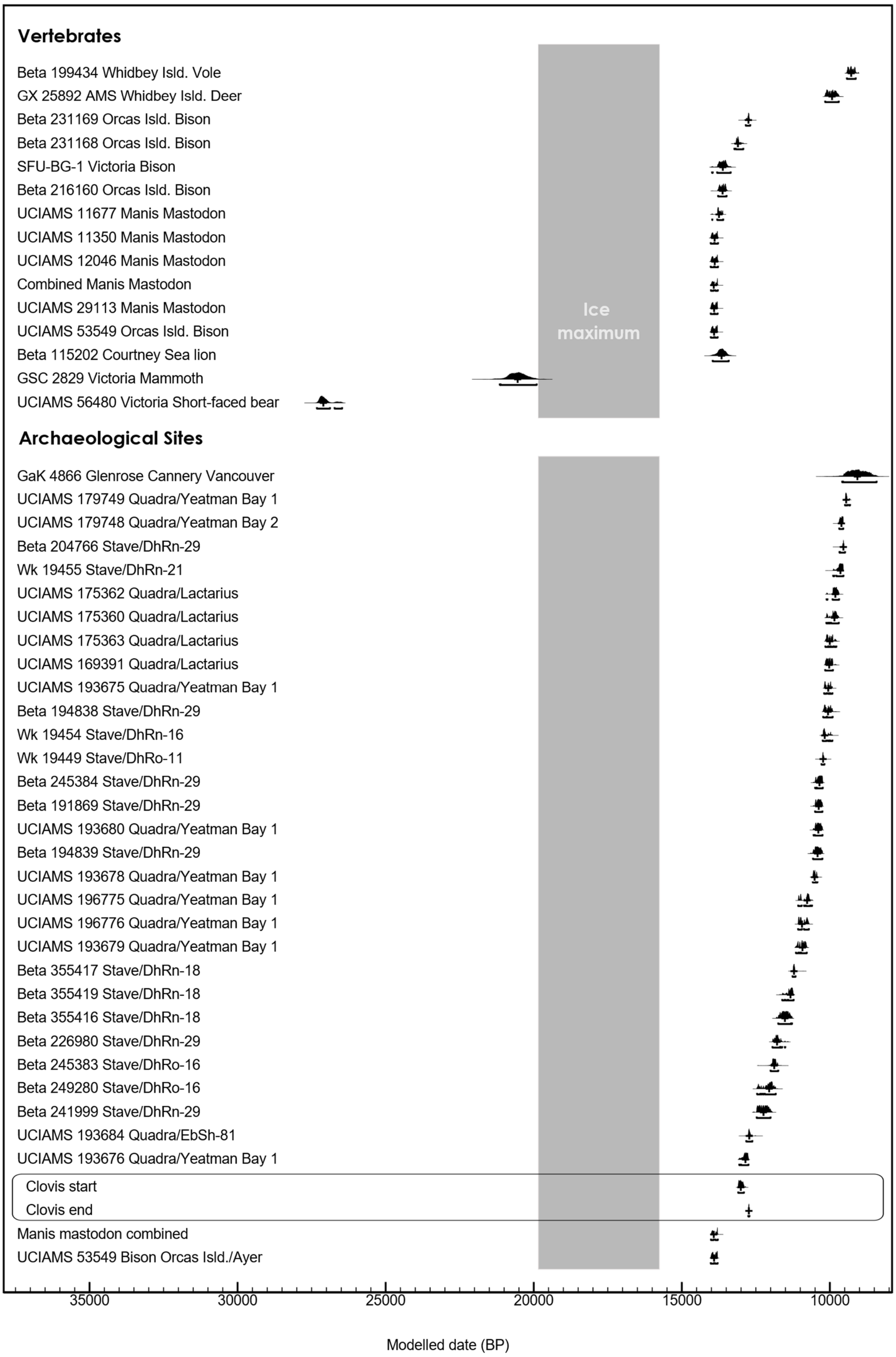

Table S1.
Table of 233 radiocarbon dates on vertebrate samples and charcoal from archaeological sites in four subregions of the north Pacific coast on which the chronologies produced in this study are based.

Southeast Alaska Subregion

**Lab Number**	**Radiocarbon Age, ± (95.4%)**	**Material sampled**	**Taxon**	**Location/Archaeological site designation**	**Reference**
Paleontology					
AA33797	10050, 100	Bone collagen	Red fox (*Vulpes vulpes*)	Alexander Archipelago	*77*
AA 32118	10020, 110	Bone collagen	Black bear (*Ursus americanus*)	Alexander Archipelago	*77*
AA 36641	10080, 120	Bone collagen	Black bear (*Ursus americanus*)	Alexander Archipelago	*77*
AA 33780	10090, 160	Bone collagen	Black bear (*Ursus americanus*)	Alexander Archipelago	*77*
CAMS 42381	10300, 50	Bone collagen	Bear (*Ursus*)	Alexander Archipelago	*77*
AA 36636	10350, 110	Bone collagen	Black bear (*Ursus americanus*)	Alexander Archipelago	*77*
AA 36640	10420, 110	Bone collagen	Black bear (*Ursus americanus*)	Alexander Archipelago	*77*
AA 18449R	10555, 110	Bone collagen	Caribou (*Rangifer tarandus*)	Alexander Archipelago	*77*
AA 15225	10970, 85	Molar collagen	Brown bear (*Ursus arctos*)	Alexander Archipelago	*77*
AA 21567	11275, 90	Bone collagen	Arctic fox (*Vulpes alopex*)	Alexander Archipelago	*77*
AA 07793	10745, 75	Bone collagen	Black bear (*Ursus americanus*)	Alexander Archipelago	*77*
AA 32120	10860, 120	Bone collagen	Black bear (*Ursus americanus*)	Alexander Archipelago	*77*
AA 32117	10870, 120	Bone collagen	Black bear (*Ursus americanus*)	Alexander Archipelago	*77*
AA 15223	11225, 110	Bone collagen	Brown bear (*Ursus arctos*)	Alexander Archipelago	*77*
AA 36638	10930, 140	Bone collagen	Black bear (*Ursus americanus*)	Alexander Archipelago	*77*
AA 32119	10970, 120	Bone collagen	Black bear (*Ursus americanus*)	Alexander Archipelago	*77*
AA 44450	11630, 120	Bone collagen	Brown bear (*Ursus arctos*)	Alexander Archipelago	*77*
AA 15222	11640, 80	Bone collagen	Brown bear (*Ursus arctos*)	Alexander Archipelago	*77*
AA 15226	11715, 120	Bone collagen	Brown bear (*Ursus arctos*)	Alexander Archipelago	*77*
AA 33202	11460, 130	Canine collagen	Black bear (*Ursus americanus*)	Alexander Archipelago	*77*
AA 10446	11540, 110	Bone collagen	Black bear (*Ursus americanus*)	Alexander Archipelago	*77*
AA 33201	11560, 100	Bone collagen	Caribou (*Rangifer tarandus*)	Alexander Archipelago	*77*
AA 10448	11565, 115	Bone collagen	Black bear (*Ursus americanus*)	Alexander Archipelago	*77*
AA 32122	11910, 140	Bone collagen	Brown bear (*Ursus arctos*)	Alexander Archipelago	*77*
AA 10445	12295, 120	Bone collagen	Brown bear (*Ursus arctos*)	Alexander Archipelago	*77*
AA 36649	12700, 140	Bone collagen	Arctic fox (*Vulpes lagopus*)	Alexander Archipelago	*77*
AA 21564	13690, 240	Bone collagen	Ringed seal (*Phoca hispida*)	Alexander Archipelago	*77*
AA 36661	14520, 470	Bone collagen	Ringed seal (*Phoca hispida*)	Alexander Archipelago	*77*
AA 37873	17130, 240	Bone collagen	Ringed seal (*Phoca hispida*)	Alexander Archipelago	*77*
AA 44445	17740, 270	Bone collagen	Ringed seal (*Phoca hispida*)	Alexander Archipelago	*77*
AA 37874	17805, 465	Bone collagen	Ringed seal (*Phoca hispida*)	Alexander Archipelago	*77*
AA 37878	18085, 230	Bone collagen	Ringed seal (*Phoca hispida*)	Alexander Archipelago	*77*
AA 36662	18770, 350	Bone collagen	Ringed seal (*Phoca hispida*)	Alexander Archipelago	*77*
AA 36658	18860, 280	Bone collagen	Ringed seal (*Phoca hispida*)	Alexander Archipelago	*77*
AA 36659	18920, 310	Bone collagen	Ringed seal (*Phoca hispida*)	Alexander Archipelago	*77*
AA 18450R	19060, 275	Bone collagen	Ringed seal (*Phoca hispida*)	Alexander Archipelago	*77*

AA 36666	19240, 260	Bone collagen	Ringed seal (*Phoca hispida*)	Alexander Archipelago	*77*
AA 33794	19170, 210	Bone collagen	Arctic fox (*Vulpes lagopus*)	Alexander Archipelago	*77*
AA 33788	19830, 350	Bone collagen	Ringed seal (*Phoca hispida*)	Alexander Archipelago	*77*
AA 33793	19480, 320	Canine collagen	Red fox (*Vulpes vulpes*)	Alexander Archipelago	*77*
AA 22884	20060, 500	Bone collagen	Ringed seal (*Phoca hispida*)	Alexander Archipelago	*77*
AA 33789	20110, 280	Bone collagen	Ringed seal (*Phoca hispida*)	Alexander Archipelago	*77*
AA 33790	20170, 450	Canine collagen	Stellar sea lion (*Eumetopias jubatus*)	Alexander Archipelago	*77*
AA 33785	20210, 270	Bone collagen	Ringed seal (*Phoca hispida*)	Alexander Archipelago	*77*
AA 36667	20300, 360	Bone collagen	Ringed seal (*Phoca hispida*)	Alexander Archipelago	*77*
AA 33197	20530, 330	Bone collagen	Velvet scooter (*Melanitta fusca*)	Alexander Archipelago	*77*
AA 36660	20470, 660	Bone collagen	Ringed seal (*Phoca hispida*)	Alexander Archipelago	*77*
AA 33784	20550, 520	Bone collagen	Ringed seal (*Phoca hispida*)	Alexander Archipelago	*77*
AA 36664	20540, 710	Bone collagen	Ringed seal (*Phoca hispida*)	Alexander Archipelago	*77*
CAMS 33980	20660, 80	Bone collagen	Ringed seal (*Phoca hispida*)	Alexander Archipelago	*77*
AA 36657	20720, 350	Bone collagen	Harbour seal (*Phoca vitulina*)	Alexander Archipelago	*77*
AA 36651	20820, 650	Premolar collagen	Stellar sea lion (*Eumetopias jubatus*)	Alexander Archipelago	*77*
AA 36668	20880, 480	Bone collagen	Ringed seal (*Phoca hispida*)	Alexander Archipelago	*77*
AA 36646	20690, 520	Bone collagen	Arctic fox (*Vulpes lagopus*)	Alexander Archipelago	*77*
AA 44444	22160, 400	Bone collagen	Ringed seal (*Phoca hispida*)	Alexander Archipelago	*77*
AA 33787	22490, 300	Bone collagen	Ringed seal (*Phoca hispida*)	Alexander Archipelago	*77*
AA 33786	23260, 470	Bone collagen	Ringed seal (*Phoca hispida*)	Alexander Archipelago	*77*
AA 37876	23315, 865	Bone collagen	Ringed seal (*Phoca hispida*)	Alexander Archipelago	*77*
AA 36645	23120, 640	Bone collagen	Red fox (*Vulpes vulpes*)	Alexander Archipelago	*77*
AA 36665	24150, 490	Bone collagen	Ringed seal (*Phoca hispida*)	Alexander Archipelago	*77*
AA 21566	23560, 770	Incisor collagen	Hoary marmot (*Marmota caligata*)	Alexander Archipelago	*77*
AA 36643	26030, 610	Bone collagen	Puffin (*Fratercula*)	Alexander Archipelago	*77*
AA33783	26820, 700	Bone collagen	Brown bear (*Ursus arctos*)	Alexander Archipelago	*77*
AA 33193	26670, 680	Bone collagen	Caribou (*Rangifer tarandus*)	Alexander Archipelago	*77*
AA 32123	27300, 1600	Bone collagen	Hoary marmot (*Marmota caligata*)	Alexander Archipelago	*77*
AA 33796	27750, 660	Bone collagen	Hoary marmot (*Marmota caligata*)	Alexander Archipelago	*77*
AA 21569	28695, 360	Bone collagen	Black bear (*Ursus americanus*)	Alexander Archipelago	*77*
AA 21570	29820, 400	Bone collagen	Black bear (*Ursus americanus*)	Alexander Archipelago	*77*
Archaeology					
CAMS 29873	9730, 60	Charcoal		49PET408, Shuká Káa / On Your Knees	*77, 78, 112*
I 6304	9130, 130	Charcoal		Ground Hog Bay 2, Component III	*114*
CAMS 32038	9880, 50	Charcoal		49PET408, Shuká Káa / On Your Knees	*77, 78, 112*
SI 2112	9220,80	Charcoal		Ground Hog Bay 2, Component III	*114*
WSU 412	10180, 800	Charcoal		Ground Hog Bay 2, Component III	*114*
CAMS 42381	10300, 50	Charcoal		49PET408, Shuká Káa / On Your Knees	*77, 78, 112*
**Haida Gwaii Subregion**					
Paleontology					
CAMS 79488	9376, 50	Bone collagen	Bear (*Ursus*)	Haida Gwaii	*81*

UCIAMS 15165	10839, 25	Bone collagen	Salmon (*Oncorhynchus*)	Haida Gwaii	*83*
UCIAMS 41042	10660, 30	Bone collagen	Brown bear (*Ursus arctos*)	Haida Gwaii	*83*
UCIAMS 15156	10715, 30	Bone collagen	Brown bear (*Ursus arctos*)	Haida Gwaii	*83*
UCIAMS 41043	10465, 30	Bone collagen	Brown bear (*Ursus arctos*)	Haida Gwaii	*83*
UCIAMS 23614	10910, 35	Bone collagen	Salmon (*Oncorhynchus*)	Haida Gwaii	*83*
UCIAMS 5754	10935, 35	Bone collagen	Salmon (*Oncorhynchus*)	Haida Gwaii	*83*
UCIAMS 15164	10970, 25	Bone collagen	Salmon (*Oncorhynchus*)	Haida Gwaii	*83*
UCIAMS 5750	10995, 35	Bone collagen	Salmon (*Oncorhynchus*)	Haida Gwaii	*83*
CAMS 79490	10450, 60	Bone collagen	Bear (*Ursus*)	Haida Gwaii	*81*
UCIAMS 21994	10465, 25	Bone collagen	Black bear (*Ursus americanus*)	Haida Gwaii	*83*
UCIAMS 5753	10485, 35	Bone collagen	Black bear (*Ursus americanus*)	Haida Gwaii	*83*
UCIAMS 5756	10515, 35	Bone collagen	Black bear (*Ursus americanus*)	Haida Gwaii	*83*
UCIAMS 5733	10550, 35	Bone collagen	Black bear (*Ursus americanus*)	Haida Gwaii	*83*
UCIAMS 15163	11510, 25	Bone collagen	Salmon (*Oncorhynchus*)	Haida Gwaii	*83*
UCIAMS 5755	10575, 35	Bone collagen	Black bear (*Ursus americanus*)	Haida Gwaii	*83*
UCIAMS 4889	10585, 45	Bone collagen	Black bear (*Ursus americanus*)	Haida Gwaii	*83*
UCIAMS 15161	10920, 35	Bone collagen	Deer (*Odocoileus hemionus*)	Haida Gwaii	*83*
UCIAMS 15159	10935, 40	Bone collagen	Deer (*Odocoileus hemionus*)	Haida Gwaii	*83*
UCIAMS 28003	10945, 25	Bone collagen	Deer (*Odocoileus hemionus*)	Haida Gwaii	*83*
UCIAMS 23611	10965, 40	Bone collagen	Deer (*Odocoileus hemionus*)	Haida Gwaii	*83*
UCIAMS 15162	10990, 25	Bone collagen	Deer (*Odocoileus hemionus*)	Haida Gwaii	*83*
UCIAMS 4892	11005, 45	Bone collagen	Deer (*Odocoileus hemionus*)	Haida Gwaii	*83*
UCIAMS 15154	11030, 30	Bone collagen	Black bear (*Ursus americanus*)	Haida Gwaii	*83*
UCIAMS 15160	11060, 30	Bone collagen	Deer (*Odocoileus hemionus*)	Haida Gwaii	*83*
CAMS 75559	11150, 50	Bone collagen	Bear (*Ursus*)	Haida Gwaii	*81*
CAMS 79489	11250, 70	Bone collagen	Bear (*Ursus*)	Haida Gwaii	*81*
CAMS 75746	14540, 70	Bone collagen	Bear (*Ursus*)	Haida Gwaii	*81*
UOC 5713	43209, 649	Bone collagen	Caribou (*Rangifer tarandus*)	White Creek	*116*
Archaeology					
CAMS 26265	8700, 60	Charcoal		1127T, Richardson Isld.	*84*
CAMS 26267	8960, 60	Charcoal		1127T, Richardson Isld.	*84*
CAMS 26266	8980, 60	Charcoal		1127T, Richardson Isld.	*84*
CAMS 26268	9080, 60	Charcoal		1127T, Richardson Isld.	*84*
CAMS 26269	9160, 60	Charcoal		1127T, Richardson Isld.	*84*
CAMS 26270	9220, 60	Charcoal		1127T, Richardson Isld.	*84*
CAMS 76668	9230, 50	Charcoal		1325T, Kilgii Gwaay	*84*
CAMS 79682	9260, 40	Charcoal		1325T, Kilgii Gwaay	*84*
CAMS 39876	9290, 50	Charcoal		1127T, Richardson Isld.	*84*
CAMS 39875	9290, 50	Charcoal		1325T, Kilgii Gwaay	*84*
CAMS 79684	9340, 40	Charcoal		1325T, Kilgii Gwaay	*84*
CAMS 87642	9395, 40	Wood wedge tool	Hemlock (*Tsuga*)	1325T, Kilgii Gwaay	*84*
CAMS 77248	9410, 50	Charcoal		1325T, Kilgii Gwaay	*84*

CAMS 87641	9415, 35	Split root tool	Spruce (*Picea*)	1325T, Kilgii Gwaay	*84*
CAMS 76666	9430, 50	Charcoal		1325T, Kilgii Gwaay	*84*
CAMS 70704	9460, 50	Bone collagen	Black bear (*Ursus americanus*)	1325T, Kilgii Gwaay	*84*
UCIAMS 49181	9485, 15	Charcoal		1906T, Gaadu Din 2	*84*
UCIAMS 49183	9530, 15	Charcoal		1906T, Gaadu Din 2	*84*
UCIAMS 12387	9930, 30	Charcoal		1693T, Gaadu Din 1	*84*
UCIAMS 12386	9980, 30	Charcoal		1693T, Gaadu Din 1	*84*
UCIAMS 55085	10025, 45	Bone collagen	Black bear (*Ursus americanus*)	1906T, Gaadu Din 2	*84*
UCIAMS 31729	10150, 25	Bone collagen (artifac	Mammalia	1693T, Gaadu Din 1	*84*
UCIAMS 55083	10205, 20	Charcoal		1906T, Gaadu Din 2	*84*
UCIAMS 55084	10210, 20	Charcoal		1906T, Gaadu Din 2	*84*
UCIAMS 49182	10215, 20	Charcoal		1906T, Gaadu Din 2	*84*
UCIAMS 40880	10220, 30	Charcoal		1906T, Gaadu Din 2	*84*
UCIAMS 56932	10280, 30	Charcoal		1906T, Gaadu Din 2	*84*
UCIAMS 40882	10295, 25	Charcoal		1906T, Gaadu Din 2	*84*
UCIAMS 56933	10530, 20	Charcoal		1906T, Gaadu Din 2	*84*
UCIAMS 12388	10550, 25	Charcoal		1906T, Gaadu Din 2	*84*
UCIAMS 4884	10510, 35	Bone collagen	Black bear (*Ursus americanus*)	FgUc-6, K1 Cave	*84*
CAMS 93774	10525, 50	Bone collagen	Black bear (*Ursus americanus*)	FgUc-6, K1 Cave	*84*
UCIAMS 28005	10615, 30	Charcoal		1693T, Gaadu Din 1	*84*
CAMS 93775	10660, 40	Bone collagen	Black bear (*Ursus americanus*)	FgUc-6, K1 Cave	*84*
UCIAMS 4886	10960, 35	Bone collagen	Black bear (*Ursus americanus*)	FgUc-6, K1 Cave	*84*
UCIAMS 40881	11030, 25	Charcoal		1906T, Gaadu Din 2	*84*
UCIAMS 28004	11665, 30	Bone collagen	Vertebrata	1693T, Gaadu Din 1	*84*
**Central Coast Subregion**					
Paleontology					
UCIAMS 56477	8660, 30	Bone collagen	Rodent (Rodentia)	Northeast Vancouver Island	*128*
Beta 10714	9830, 140	Bone collagen	Black bear (*Ursus americanus*)	Northeast Vancouver Island	*130*
UCIAMS 56478	10100, 30	Bone collagen	Small mammal (Rodentia)	Northeast Vancouver Island	91
UCIAMS 59635	10535, 50	Bone collagen	Vertebrata	Northeast Vancouver Island	*128*
UCIAMS 41052	11110, 30	Bone collagen	Brown bear (*Ursus arctos*)	Northeast Vancouver Island	*90*
UCIAMS 118263	11170, 30	Bone collagen	Cricetidae	Northeast Vancouver Island	*91*
UCIAMS 118261	11460, 60	Bone collagen	Cricetidae	Northeast Vancouver Island	*91*
UCIAMS 118259	11490, 60	Bone collagen	Cricetidae	Northeast Vancouver Island	*91*
UCIAMS 41049	11615, 30	Bone collagen	Short-faced bear (*Arctodus simus*)	Northeast Vancouver Island	*90*
OxA 24005	11720, 50	Molar collagen	Short-faced bear (*Arctodus simus*)	Northeast Vancouver Island	*90*
UCIAMS 41048	11775, 30	Bone collagen	Short-faced bear (*Arctodus simus*)	Northeast Vancouver Island	*90*
UCIAMS 118260	11840, 90	Bone collagen	Cricetidae	Northeast Vancouver Island	*91*
UCIAMS 56479	11935, 40	Bone collagen	Black bear (*Ursus americanus*)	Northeast Vancouver Island	*90*
UCIAMS 73321	11960, 45	Bone collagen	Cricetidae	Northeast Vancouver Island	*128*
TO 5006	12070, 70	Bone collagen	Mountain Goat (*Oreamnos americanus*)	Northeast Vancouver Island	*129*
UCIAMS 73320	12110, 45	Bone collagen	Bear (*Ursus*)	Northeast Vancouver Island	*128*

TO 6072	12200, 190	Bone collagen	Mountain Goat (*Oreamnos americanus*)	Northeast Vancouver Island	*129*
CAMS 97342	12340, 50	Bone collagen	Mountain Goat (*Oreamnos americanus*)	Northwest Vancouver Island	*92*
UICAMS 118265	12370, 35	Bone collagen	Brown bear (*Ursus arctos*)	Northeast Vancouver Island	*128*
UICAMS 41050	12425, 35	Bone collagen	Brown bear (*Ursus arctos*)	Northeast Vancouver Island	*90*
UCIAMS 41051	12440, 35	Bone collagen	Brown bear (*Ursus arctos*)	Northeast Vancouver Island	*90*
CAMS 88275	16270, 170	Bone collagen	Sparrow (cf. *Passer*)	Northwest Vancouver Island	*67*
CAMS 74625	16340, 60	Bone collagen	Vole (*Arvicolinae*)	Northwest Vancouver Island	*67*
CAMS 102798	16340, 60	Bone collagen	Mountain Goat (*Oreamnos americanus*)	Northwest Vancouver Island	*92*
CAMS 88274	16460, 170	Bone collagen	Marmot (*Marmota*)	Northwest Vancouver Island	*67*
CAMS 97341	16965, 45	Bone collagen	Vertebrata	Northwest Vancouver Island	*92*
CAMS 102797	17100, 70	Bone collagen	Vole (*Arvicolinae*)	Northwest Vancouver Island	*92*
CAMS 74624	18010, 100	Bone collagen	Vole (*Arvicolinae*)	Northwest Vancouver Island	*67*
Archaeology					
WSU-2141/1978	8020, 110	Charcoal		EeSu-8, Bear Cove	*126*
WAT 519	9000, 140	Charcoal		ElSx-1, Namu	*121*
UICAMS 112263	9140, 25	Charcoal		EkTb-9, Triquet Isld.	*125*
GAK 3244	9140, 200	Charcoal		ElSx-1, Namu	*121*
UCIAMS 61764	9345, 25	Charcoal		EdSr-42, Kokish River	*91*
WAT 452	9720, 140	Charcoal		ElSx-1, Namu	*121*
UCIAMS 163730	9845, 25	Charcoal		EkTb-9, Triquet Isld.	*125*
UCIAMS 118001	9960, 25	Charcoal		EkTb-9, Triquet Isld.	*125*
Beta 109626	9940,50	Charcoal		ElTa-18, Hunter Isld.	*123*
UCIAMS 184916	10625, 20	Wood	Pine (*Pinus*)	EjTa-4, Calvert Isld.	*124*
UCIAMS 163740	10720, 60	Wood	Pine (*Pinus*)	EjTa-4, Calvert Isld.	*124*
UCIAMS 169399	10980, 25	Wood	Pine (*Pinus*)	EjTa-4, Calvert Isld.	*124*
UCIAMS 163741	11260, 25	Wood	Pine (*Pinus*)	EjTa-4, Calvert Isld.	*124*
UCIAMS 142561	11295, 30	Wood		EjTa-4, Calvert Isld.	*124*
UCIAMS 169402	11365, 25	Wood	Pine (*Pinus*)	EjTa-4, Calvert Isld.	*124*
UCIAMS 149779	11435, 30	Wood		EjTa-4, Calvert Isld.	*124*
UCIAMS 163739	11440, 25	Wood	Pine (*Pinus*)	EjTa-4, Calvert Isld.	*124*
UCIAMS 179732	12010, 180	Charcoal		EkTb-9, Triquet Isld.	*125*
**South Coast Subregion**					
Paleontology					
Beta 199434	8280, 40	Bone collagen	Vole (*Arvicolinae*)	Whidbey Isld.	*140*
GX-25892-AMS	8840, 50	Bone collagen	Deer (*Odocoileus hemionus*)	Whidbey Isld.	*140*
Beta 231169	10800, 60	Bone collagen	Bison (*Bison antiquus*)	Orcas Isld.	*101*
Beta 231168	11180, 60	Bone collagen	Bison (*Bison antiquus*)	Orcas Isld.	*101*
SFU-BG-1	11750, 110	Bone collagen	Bison (*Bison*)	North Saanich	*139*
Beta 216160	11760, 70	Bone collagen	Bison (*Bison antiquus*)	Orcas Isld.	*101*
UCIAMS 11677	11890, 35	Ivory collagen	Mastodon (*Mammut*)	Squim, Washington	*69*
UCIAMS 11350	11975, 35	Ivory collagen	Mastodon (*Mammut*)	Squim, Washington	*69*
UCIAMS 12046	11975, 35	Ivory collagen	Mastodon (*Mammut*)	Squim, Washington	*69*

Combined Manis Mastodon	11969, 18	XAD-Collagen	Mastodon (*Mammut*)	Squim, Washington	*69*
UCIAMS 29113	11990, 30	Bone collagen	Mastodon (*Mammut*)	Squim, Washington	*69*
UCIAMS 53549	11990, 25	Bone collagen	Bison (*Bison antiquus*)	Ayer/Orcas Isld.	*70*
Beta 115202	12570, 70	Bone collagen	Stellar sea lion (*Eumetopias jubatus*)	Courtney	*100*
GSC 2829	17000, 240	Bone collagen	Mammoth (*Mammuthus*)	Victoria	*138*
UCIAMS 56480	22750, 140	Bone collagen	Giant short-faced bear (*Arctodus simus*)	Cowichan Head	*99*
Archaeology					
GaK 4866	8150, 250	Charcoal		DgRr-6, Glenrose Cannery	*133*
UCIAMS 179749	8405, 20	Charcoal		EbSh-98, Yeatman Bay 1, Quadra Isld.	*131*
UCIAMS 179748	8680, 20	Charcoal		EbSh-98, Yeatman Bay 2, Quadra Isld.	*131*
Beta 204766	8590, 40	Charcoal		DhRn-29, Stave Lake	*103*
Wk 19455	8702, 43	Charcoal		DhRn-21, Stave Lake	*103*
UCIAMS 175362	8800, 20	Charcoal		EaSh-81, Lactarius Road, Quadra Isld.	*35, 131*
UCIAMS 175360	8820, 25	Charcoal		EaSh-81, Lactarius Road, Quadra Isld.	*35, 131*
UCIAMS 175363	8855, 20	Charcoal		EaSh-81, Lactarius Road, Quadra Isld.	*35, 131*
UCIAMS 169391	8875, 20	Charcoal		EaSh-81, Lactarius Road, Quadra Isld.	*35, 131*
UCIAMS 193675	8935, 20	Charcoal		EbSh-98, Yeatman Bay 1, Quadra Isld.	*131*
Beta 194838	8950, 50	Charcoal		DhRn-29, Stave Lake	*103*
Wk 19454	8990, 46	Charcoal		DhRn-16, Stave Lake	*103*
Wk 19449	9075, 34	Charcoal		DhRo-11, Stave Lake	*103*
Beta 245384	9190, 50	Charcoal		DhRn-29, Stave Lake	*103*
Beta 191869	9220, 40	Charcoal		DhRn-29, Stave Lake	*103*
UCIAMS 193680	9230,45	Charcoal		EbSh-98, Yeatman Bay 1, Quadra Isld.	*131*
Beta 194839	9250, 60	Charcoal		DhRn-29, Stave Lake	*103*
UCIAMS 193678	9305, 20	Charcoal		EbSh-98, Yeatman Bay 1, Quadra Isld.	*131*
UCIAMS 196775	9505, 30	Charcoal		EbSh-98, Yeatman Bay 1, Quadra Isld.	*131*
UCIAMS 196776	9540, 25	Charcoal		EbSh-98, Yeatman Bay 1, Quadra Isld.	*131*
UCIAMS 193679	9605, 20	Charcoal		EbSh-98, Yeatman Bay 1, Quadra Isld.	*131*
Beta 355417	9780, 40	Charcoal		DhRn-18, Stave Lake	*103*
Beta 355419	9920, 50	Charcoal		DhRn-18, Stave Lake	*103*
Beta 355416	10020, 50	Charcoal		DhRn-18, Stave Lake	*103*
Beta 226980	10150, 40	Charcoal		DhRn-29, Stave Lake	*103*
Beta 245383	10210, 40	Charcoal		DhRo-16, Stave Lake	*103*
Beta 249280	10290, 50	Charcoal		DhRo-16, Stave Lake	*103*
Beta 241999	10370, 40	Charcoal		DhRn-29, Stave Lake	*103*
UCIAMS 193684	10740, 70	Charcoal		EbSh-81, Crescent Road, Quadra Isld.	*131*
UCIAMS 193676	10940, 60	Charcoal		EbSh-98, Yeatman Bay 1, Quadra Isld.	*131*
Clovis start	11080, 40	Various		Clovis	*17*
Clovis end	10765, 25	Bone collagen	Bison (*Bison*)	Clovis	*17*
Manis mastodon combined	11969, 18	XAD-Collagen	Mastodon (*Mammut*)	45CA218, Manis Mastodon, Squim, Washington	*68, 69*
UCIAMS 53549	11990, 25	Bone collagen	Bison (*Bison antiquus*)	45SJ454.1, Ayer / Orcas Isld.	*70*

Table S2.

Radiocarbon date calibrations and code for the 74 age estimates that comprised the southeast Alaska chronology. Source references for each date are in table S1. A: Calibrated radiocarbon age estimates in calendar years befor present (BP), B: Code from OxCal 4.4.4.

**A. Southeast Alaska Subregion (n=74)**	Unmodelled (BP)			Modelled (BP)		
	median	from_95_4	to_95_4	median	from_95_4	to_95_4
IntCal20						
Marine20						
LocalMarine1	257	58.5	455.5	274	76	470
Mixed1	60	59.2	60.8	60	58.7	61.3
AA33797 Red fox	10972	11255	10606	10970	11255	10602
IntCal20						
AA 32118 Black bear	11544	11928	11239	11544	11929	11238
AA 36641 Black bear	11633	12097	11238	11633	12097	11237
AA 33780 Black bear	11682	12456	11225	11682	12456	11224
CAMS 42381 Bear	12079	12462	11833	12079	12462	11833
AA 36636 Black bear	12199	12616	11815	12199	12616	11815
AA 36640 Black bear	12289	12678	11935	12289	12678	11934
AA 18449R Caribou	12526	12739	12060	12526	12740	12061
IntCal20						
Marine20						
LocalMarine1	257	58.5	455.5	257	58	455.5
Mixed1	40	39.2	40.8	40	38.5	41.5
AA 15225 Brown bear	12630	12763	12335	12630	12764	12336
IntCal20						
Marine20						
LocalMarine1	257	58.5	455.5	257	59	455.5
Mixed1	86	85.2	86.8	86	84.5	87.5
AA 21567 Arctic fox	12500	12742	12104	12503	12745	12109
IntCal20						
AA 07793 Black bear	12724	12833	12514	12724	12833	12514

AA 32120 Black bear	12825	13085	12678	12825	13085	12677
AA 32117 Black bear	12833	13084	12687	12833	13084	12687
IntCal20						
Marine20						
LocalMarine1	257	58.5	455.5	257	58	458
Mixed1	40	39.2	40.8	40	38.5	41.5
AA 15223 Brown bear	12843	13081	12686	12835	13079	12678
IntCal20						
AA 36638 Black bear	12886	13114	12695	12886	13112	12695
AA 32119 Black bear	12908	13097	12739	12908	13097	12738
IntCal20						
Marine20						
LocalMarine1	257	58.5	455.5	257	58.5	455.5
Mixed1	40	39.2	40.8	40	38.5	41.5
AA 44450 Brown bear	13210	13462	12937	13204	13457	12935
AA 15222 Brown bear	13214	13404	13080	13208	13402	13073
AA 15226 Brown bear	13283	13572	13080	13277	13572	13075
IntCal20						
AA 33202 Black bear	13337	13585	13110	13337	13585	13110
AA 10446 Black bear	13404	13597	13179	13404	13597	13180
AA 33201 Caribou	13421	13602	13185	13421	13603	13185
AA 10448 Black bear	13427	13735	13178	13427	13735	13178
IntCal20						
Marine20						
LocalMarine1	257	58.5	455.5	257	58	454
Mixed1	40	39.2	40.8	40	38.5	41.5
AA 32122 Brown bear	13471	13773	13178	13464	13770	13176
AA 10445 Brown bear	13897	14215	13526	13890	14199	13523
IntCal20						
Marine20						
LocalMarine1	257	58.5	455.5	257	58	456
Mixed1	86	85.2	86.8	86	84.5	87.5

AA 36649 Arctic fox	13996	14585	13510	13968	14540	13498
Marine20						
LocalMarine	257	58.5	455.5	274.5	75	472
AA 21564 Ringed seal	15343	16098	14531	15323	16076	14501
AA 36661 Ringed seal	16439	17730	15222	16420	17704	15203
AA 37873 Ringed seal	19474	20142	18838	19453	20121	18821
AA 44445 Ringed seal	20209	20924	19472	20191	20909	19461
AA 37874 Ringed seal	20315	21592	19151	20294	21566	19132
AA 37878 Ringed seal	20627	21324	19986	20605	21298	19964
AA 36662 Ringed seal	21482	22340	20584	21465	22325	20563
AA 36658 Ringed seal	21589	22301	20837	21573	22285	20820
AA 36659 Ringed seal	21657	22414	20831	21641	22405	20821
AA 18450R Ringed seal	21827	22499	21029	21812	22480	21014
AA 36666 Ringed seal	22043	22732	21331	22028	22717	21316
IntCal20						
Marine20						
LocalMarine1	257	58.5	455.5	257	58.5	456
Mixed1	86	85.2	86.8	86	84.5	87.5
AA 33794 Arctic fox	22153	22715	21560	22142	22695	21532
Marine20						
LocalMarine	257	58.5	455.5	256.5	58.5	455
AA 33788 Ringed seal	22692	23610	21945	22692	23610	21943
IntCal20						
Marine20						
LocalMarine1	257	58.5	455.5	257	59	456.5
Mixed1	60	59.2	60.8	60	58.7	61.3
AA 33793 Red fox	22788	23673	22160	22778	23670	22141
Marine20						
LocalMarine	257	58.5	455.5	270.5	65	462.5
AA 22884 Ringed seal	22966	24165	21867	22953	24164	21855
AA 33789 Ringed seal	22990	23700	22351	22975	23690	22343
AA 33790 Sea lion	23079	24135	22089	23067	24129	22078

AA 33785 Ringed seal	23104	23750	22445	23091	23740	22439
AA 36667 Ringed seal	23208	24040	22370	23197	24040	22365
AA 33197 Velvet scooter	23442	24255	22655	23431	24240	22636
AA 36660 Ringed seal	23444	25069	22043	23433	25031	22017
AA 33784 Ringed seal	23498	24782	22355	23486	24779	22338
AA 36664 Ringed seal	23531	25261	22023	23522	25253	22013
CAMS 33980 Ringed seal	23569	23891	23175	23576	23902	23184
AA 36657 Harbor seal	23651	24568	22858	23639	24551	22846
AA 36651 Sea lion	23829	25370	22409	23816	25342	22401
AA 36668 Ringed seal	23866	25074	22808	23852	25056	22794
IntCal20						
Marine20						
LocalMarine1	257	58.5	455.5	257	58.5	454.5
Mixed1	86	85.2	86.8	86	84.5	87.5
AA 36646 Arctic fox	23839	25132	22696	23823	25105	22669
Marine20						
LocalMarine	257	58.5	455.5	262.5	63	463
AA 44444 Ringed seal	25272	26133	24292	25269	26138	24285
AA 33787 Ringed seal	25608	26298	24941	25605	26292	24939
AA 33786 Ringed seal	26413	27380	25485	26409	27380	25489
AA 37876 Ringed seal	26513	28485	24770	26508	28448	24769
IntCal20						
Marine20						
LocalMarine1	257	58.5	455.5	257	58.5	455.5
Mixed1	60	59.2	60.8	60	58.7	61.3
AA 36645 Red fox	26748	28109	25548	26740	28094	25545
Marine20						
LocalMarine	257	58.5	455.5	257.5	58	457
AA 36665 Ringed seal	27275	28395	26245	27276	28386	26238
IntCal20						
AA 21566 Marmot	27901	29666	26373	27901	29649	26372
Marine20						

LocalMarine	257	58.5	455.5	257.5	59	457.5
AA 36643 Puffin	29177	30434	27839	29178	30426	27833
IntCal20						
Marine20						
LocalMarine1	257	58.5	455.5	257	58	456
Mixed1	40	39.2	40.8	40	38.5	41.5
AA33783 Brown bear	30619	32065	29170	30615	32057	29170
IntCal20						
AA 33193 Caribou	30858	32785	29322	30858	32787	29337
AA 32123 Marmot	31989	36172	28706	31987	36137	28716
AA 33796 Marmot	32015	33832	30866	32016	33833	30865
AA 21569 Black bear	33000	34020	31931	33000	34015	31929
AA 21570 Black bear	34325	35221	33492	34326	35227	33492
Marine20						
LocalMarine	257	58.5	455.5	266.5	54.5	468.5
CAMS 29873 49PET408	10146	10498	9766	10146	10499	9767
IntCal20						
I 6304 Ground Hog Bay III	10321	10655	9905	10321	10656	9905
Marine20						
LocalMarine	257	58.5	455.5	245.5	50	455.5
CAMS 32038 49PET408	10351	10690	10030	10352	10690	10030
IntCal20						
SI 2112 Ground Hog Bay III	10392	10577	10235	10392	10578	10235
WSU 412 Ground Hog Bay III	11842	13806	9604	11840	13807	9671
CAMS 42381 49PET408	12079	12462	11833	12079	12462	11833

B. OXCAL CODE

```
Plot(Southeast Alaska Subregion)
 {
  Curve("IntCal20","intcal20.14c");
  Curve("Marine20","marine20.14c");
```

```
Delta_R("LocalMarine1",257,99);
Mix_Curves("Mixed1","IntCal20","LocalMarine1",60,0.23);
R_Date("AA33797 Red fox",10050,100);
Curve("IntCal20","intcal20.14c");
R_Date("AA 32118 Black bear",10020,110);
R_Date("AA 36641 Black bear",10080,120);
R_Date("AA 33780 Black bear",10090,160);
R_Date("CAMS 42381 Bear",10300,50);
R_Date("AA 36636 Black bear",10350,110);
R_Date("AA 36640 Black bear",10420,110);
R_Date("AA 18449R Caribou",10555,110);
Curve("IntCal20","intcal20.14c");
Curve("Marine20","marine20.14c");
Delta_R("LocalMarine1",257,99);
Mix_Curves("Mixed1","IntCal20","LocalMarine1",40,0.18);
R_Date("AA 15225 Brown bear",10970,85);
Curve("IntCal20","intcal20.14c");
Curve("Marine20","marine20.14c");
Delta_R("LocalMarine1",257,99);
Mix_Curves("Mixed1","IntCal20","LocalMarine1",86,0.10);
R_Date("AA 21567 Arctic fox",11275,90);
Curve("IntCal20","intcal20.14c");
R_Date("AA 07793 Black bear",10745,75);
R_Date("AA 32120 Black bear",10860,120);
R_Date("AA 32117 Black bear",10870,120);
Curve("IntCal20","intcal20.14c");
Curve("Marine20","marine20.14c");
Delta_R("LocalMarine1",257,99);
Mix_Curves("Mixed1","IntCal20","LocalMarine1",40,0.18);
R_Date("AA 15223 Brown bear",11225,110);
Curve("IntCal20","intcal20.14c");
R_Date("AA 36638 Black bear",10930,140);
```

```
R_Date("AA 32119 Black bear",10970,120);
Curve("IntCal20","intcal20.14c");
Curve("Marine20","marine20.14c");
Delta_R("LocalMarine1",257,99);
Mix_Curves("Mixed1","IntCal20","LocalMarine1",40,0.18);
R_Date("AA 44450 Brown bear",11630,120);
R_Date("AA 15222 Brown bear",11640,80);
R_Date("AA 15226 Brown bear",11715,120);
Curve("IntCal20","intcal20.14c");
R_Date("AA 33202 Black bear",11460,130);
R_Date("AA 10446 Black bear",11540,110);
R_Date("AA 33201 Caribou",11560,100);
R_Date("AA 10448 Black bear",11565,115);
Curve("IntCal20","intcal20.14c");
Curve("Marine20","marine20.14c");
Delta_R("LocalMarine1",257,99);
Mix_Curves("Mixed1","IntCal20","LocalMarine1",40,0.18);
R_Date("AA 32122 Brown bear",11910,140);
R_Date("AA 10445 Brown bear",12295,120);
Curve("IntCal20","intcal20.14c");
Curve("Marine20","marine20.14c");
Delta_R("LocalMarine1",257,99);
Mix_Curves("Mixed1","IntCal20","LocalMarine1",86,0.10);
R_Date("AA 36649 Arctic fox",12700,140);
Curve("Marine20","marine20.14c");
Delta_R("LocalMarine",257,99);
R_Date("AA 21564 Ringed seal",13690,240);
R_Date("AA 36661 Ringed seal",14520,470);
R_Date("AA 37873 Ringed seal",17130,240);
R_Date("AA 44445 Ringed seal",17740,270);
R_Date("AA 37874 Ringed seal",17805,465);
R_Date("AA 37878 Ringed seal",18085,230);
```

```
R_Date("AA 36662 Ringed seal",18770,350);
R_Date("AA 36658 Ringed seal",18860,280);
R_Date("AA 36659 Ringed seal",18920,310);
R_Date("AA 18450R Ringed seal",19060,275);
R_Date("AA 36666 Ringed seal",19240,260);
Curve("IntCal20","intcal20.14c");
Curve("Marine20","marine20.14c");
Delta_R("LocalMarine1",257,99);
Mix_Curves("Mixed1","IntCal20","LocalMarine1",86,0.10);
R_Date("AA 33794 Arctic fox",19170,210);
Curve("Marine20","marine20.14c");
Delta_R("LocalMarine",257,99);
R_Date("AA 33788 Ringed seal",19830,350);
Curve("IntCal20","intcal20.14c");
Curve("Marine20","marine20.14c");
Delta_R("LocalMarine1",257,99);
Mix_Curves("Mixed1","IntCal20","LocalMarine1",60,0.23);
R_Date("AA 33793 Red fox",19480,320);
Curve("Marine20","marine20.14c");
Delta_R("LocalMarine",257,99);
R_Date("AA 22884 Ringed seal",20060,500);
R_Date("AA 33789 Ringed seal",20110,280);
R_Date("AA 33790 Sea lion",20170,450);
R_Date("AA 33785 Ringed seal",20210,270);
R_Date("AA 36667 Ringed seal",20300,360);
R_Date("AA 33197 Velvet scooter",20530,330);
R_Date("AA 36660 Ringed seal",20470,660);
R_Date("AA 33784 Ringed seal",20550,520);
R_Date("AA 36664 Ringed seal",20540,710);
R_Date("CAMS 33980 Ringed seal",20660,80);
R_Date("AA 36657 Harbor seal",20720,350);
R_Date("AA 36651 Sea lion",20820,650);
```

```
R_Date("AA 36668 Ringed seal",20880,480);
Curve("IntCal20","intcal20.14c");
Curve("Marine20","marine20.14c");
Delta_R("LocalMarine1",257,99);
Mix_Curves("Mixed1","IntCal20","LocalMarine1",86,0.10);
R_Date("AA 36646 Arctic fox",20690,520);
Curve("Marine20","marine20.14c");
Delta_R("LocalMarine",257,99);
R_Date("AA 44444 Ringed seal",22160,400);
R_Date("AA 33787 Ringed seal",22490,300);
R_Date("AA 33786 Ringed seal",23260,470);
R_Date("AA 37876 Ringed seal",23315,865);
Curve("IntCal20","intcal20.14c");
Curve("Marine20","marine20.14c");
Delta_R("LocalMarine1",257,99);
Mix_Curves("Mixed1","IntCal20","LocalMarine1",60,0.23);
R_Date("AA 36645 Red fox",23120,640);
Curve("Marine20","marine20.14c");
Delta_R("LocalMarine",257,99);
R_Date("AA 36665 Ringed seal",24150,490);
Curve("IntCal20","intcal20.14c");
R_Date("AA 21566 Marmot",23560,770);
Curve("Marine20","marine20.14c");
Delta_R("LocalMarine",257,99);
R_Date("AA 36643 Puffin",26030,610);
Curve("IntCal20","intcal20.14c");
Curve("Marine20","marine20.14c");
Delta_R("LocalMarine1",257,99);
Mix_Curves("Mixed1","IntCal20","LocalMarine1",40,0.18);
R_Date("AA33783 Brown bear",26820,700);
Curve("IntCal20","intcal20.14c");
R_Date("AA 33193 Caribou",26670,680);
```

```
 R_Date("AA 32123 Marmot",27300,1600);
 R_Date("AA 33796 Marmot",27750,660);
 R_Date("AA 21569 Black bear",28695,360);
 R_Date("AA 21570 Black bear",29820,400);
 Curve("Marine20","marine20.14c");
 Delta_R("LocalMarine",257,99);
 R_Date("CAMS 29873 49PET408",9730,60);
 Curve("IntCal20","intcal20.14c");
 R_Date("I 6304 Ground Hog Bay III",9130,130);
 Curve("Marine20","marine20.14c");
 Delta_R("LocalMarine",257,99);
 R_Date("CAMS 32038 49PET408",9880,50);
 Curve("IntCal20","intcal20.14c");
 R_Date("SI 2112 Ground Hog Bay III",9220,80);
 R_Date("WSU 412 Ground Hog Bay III",10180,800);
 R_Date("CAMS 42381 49PET408",10300,50);
};
```

Table S3.

Radiocarbon date calibrations and code for the 66 age estimates that comprised the Haida Gwaii chronology. Source references are in table S1. A: Calibrated radiocarbon age estimates in calendar years befor present (BP), B: Code from OxCal 4.4.4.

**A. Haida Gwaii Subregion (n=66)**	Unmodelled (BP)			Modelled (BP)		
	median	from_95_4	to_95_4	median	from_95_4	to_95_4
CAMS 79488 Bear	10600	10737	10434	10600	10738	10434
Marine20						
LocalMarine	257	58.5	455.5	265	59	454
UCIAMS 15165 Salmon	11689	12054	11295	11690	12064	11300
IntCal20						
Marine20						
LocalMarine1	257	58.5	455.5	283	54	504
Mixed1	65	64.5	65.5	65	64.5	65.5
UCIAMS 41042 Brown bear	11836	12103	11508	11814	12103	11494
IntCal20						
Marine20						
LocalMarine1	257	58.5	455.5	257.5	58	455.5
Mixed1	78	77.5	78.5	78	77.5	78.5
UCIAMS 15156 Brown bear	11761	12055	11401	11741	12084	11388
IntCal20						
Marine20						
LocalMarine1	257	58.5	455.5	257	59	455.5
Mixed1	39	38.5	39.5	39	38.5	39.5
UCIAMS 41043 Brown bear	11839	11998	11644	11826	11996	11626
Marine20						
LocalMarine	257	58.5	455.5	237.5	48.5	468.5
UCIAMS 23614 Salmon	11806	12231	11389	11832	12249	11392
UCIAMS 5754 Salmon	11848	12265	11430	11870	12311	11427
UCIAMS 15164 Salmon	11906	12323	11508	11923	12364	11499
UCIAMS 5750 Salmon	11948	12382	11570	11963	12411	11573

IntCal20						
CAMS 79490 Bear	12350	12620	12055	12350	12620	12056
UCIAMS 21994 Black bear	12547	12614	12196	12547	12615	12195
UCIAMS 5753 Black bear	12548	12623	12195	12548	12624	12195
UCIAMS 5756 Black bear	12550	12673	12470	12550	12672	12470
UCIAMS 5733 Black bear	12576	12684	12480	12577	12684	12481
Marine20						
LocalMarine	257	58.5	455.5	271	54	478
UCIAMS 15163 Salmon	12609	12832	12338	12610	12836	12337
IntCal20						
UCIAMS 5755 Black bear	12623	12697	12487	12623	12696	12487
UCIAMS 4889 Black bear	12630	12712	12486	12630	12713	12486
UCIAMS 15161 Deer	12812	12895	12755	12812	12895	12755
UCIAMS 15159 Deer	12829	12957	12751	12829	12957	12751
UCIAMS 28003 Deer	12836	12901	12763	12836	12901	12762
UCIAMS 23611 Deer	12868	13054	12760	12868	13055	12760
UCIAMS 15162 Deer	12891	13062	12826	12891	13062	12826
UCIAMS 4892 Deer	12929	13080	12785	12929	13080	12785
UCIAMS 15154 Black bear	12965	13076	12843	12965	13076	12843
UCIAMS 15160 Deer	12997	13087	12902	12997	13087	12902
CAMS 75559 Bear	13082	13164	12926	13082	13165	12925
CAMS 79489 Bear	13154	13305	13076	13154	13305	13075
IntCal20						
Marine20						
LocalMarine1	257	58.5	455.5	257	58	454
Mixed1	30	29.5	30.5	30	29.5	30.5
CAMS 75746 Bear	17311	17540	17065	17306	17534	17062
IntCal20						
UOC 5713 Caribou	45669	47006	44605	45668	47006	44608
CAMS 26265 Richardson Isld.	9663	9893	9541	9663	9892	9541
CAMS 26267 Richardson Isld.	10071	10235	9904	10071	10235	9903
CAMS 26266 Richardson Isld.	10129	10241	9908	10129	10241	9908

CAMS 26268 Richardson Isld.	10239	10486	9970	10239	10487	9970
CAMS 26269 Richardson Isld.	10333	10499	10225	10333	10499	10225
CAMS 26270 Richardson Isld.	10384	10560	10244	10383	10560	10243
CAMS 76668 Kilgii Gwaay	10391	10555	10249	10391	10556	10249
CAMS 79682 Kilgii Gwaay	10433	10568	10286	10433	10568	10287
CAMS 39876 Richardson Isld.	10472	10649	10284	10472	10649	10284
CAMS 39875 Richardson Isld.	10472	10649	10284	10472	10649	10284
CAMS 79684 Kilgii Gwaay	10548	10687	10420	10549	10687	10421
CAMS 87642 Kilgii Gwaay	10624	10726	10507	10624	10725	10507
CAMS 77248 Kilgii Gwaay	10637	10990	10502	10637	10990	10501
CAMS 87641 Kilgii Gwaay	10640	10745	10519	10640	10745	10520
CAMS 76666 Kilgii Gwaay	10660	11060	10510	10660	11060	10509
CAMS 70704 Kilgii Gwaay	10701	11069	10565	10701	11069	10565
UCIAMS 49181 GD2	10726	11060	10602	10726	11060	10603
UCIAMS 49183 GD2	10960	11069	10707	10961	11069	10707
UCIAMS 12387 GD1	11319	11601	11242	11319	11600	11241
UCIAMS 12386 GD1	11420	11682	11269	11419	11681	11269
UCIAMS 55085 GD2	11526	11748	11311	11526	11747	11311
UCIAMS 31729 GD1	11788	11931	11648	11788	11931	11648
UCIAMS 55083 GD2	11877	11942	11765	11877	11943	11765
UCIAMS 55084 GD2	11881	11944	11767	11881	11945	11766
UCIAMS 49182 GD2	11885	11946	11817	11886	11947	11817
UCIAMS 40880 GD2	11889	11995	11761	11889	11994	11760
UCIAMS 56932 GD2	11991	12432	11829	11991	12433	11828
UCIAMS 40882 GD2	12018	12438	11885	12017	12439	11884
UCIAMS 56933 GD2	12537	12663	12479	12537	12662	12479
UCIAMS 12388 GD1	12539	12675	12485	12539	12675	12485
UCIAMS 4884 K1	12551	12670	12334	12550	12671	12334
CAMS 93774 K1	12553	12692	12201	12553	12692	12203
UCIAMS 28005 GD1	12656	12715	12501	12657	12715	12501
CAMS 93775 K1	12691	12734	12617	12691	12734	12617
UCIAMS 4886 K1	12860	12990	12758	12860	12985	12759

UCIAMS 40881 GD2	12965	13074	12844	12965	13074	12844
UCIAMS 28004 GD1	13539	13595	13461	13539	13595	13460

B. OXCAL CODE

```
Plot(Haida Gwaii Subregion)
{
 R_Date("CAMS 79488 Bear",9376,50);
 Curve("Marine20","marine20.14c");
 Delta_R("LocalMarine",257,99);
 R_Date("UCIAMS 15165 Salmon",10839,25);
 Curve("IntCal20","intcal20.14c");
 Curve("Marine20","marine20.14c");
 Delta_R("LocalMarine1",257,99);
 Mix_Curves("Mixed1","IntCal20","LocalMarine1",65,0.003);
 R_Date("UCIAMS 41042 Brown bear",10660,30);
 Curve("IntCal20","intcal20.14c");
 Curve("Marine20","marine20.14c");
 Delta_R("LocalMarine1",257,99);
 Mix_Curves("Mixed1","IntCal20","LocalMarine1",78,0.004);
 R_Date("UCIAMS 15156 Brown bear",10715,30);
 Curve("IntCal20","intcal20.14c");
 Curve("Marine20","marine20.14c");
 Delta_R("LocalMarine1",257,99);
 Mix_Curves("Mixed1","IntCal20","LocalMarine1",39,0.004);
 R_Date("UCIAMS 41043 Brown bear",10465,30);
 Curve("Marine20","marine20.14c");
 Delta_R("LocalMarine",257,99);
 R_Date("UCIAMS 23614 Salmon",10910,35);
 R_Date("UCIAMS 5754 Salmon",10935,35);
 R_Date("UCIAMS 15164 Salmon",10970,25);
 R_Date("UCIAMS 5750 Salmon",10995,35);
```

```
Curve("IntCal20","intcal20.14c");
R_Date("CAMS 79490 Bear",10450,60);
R_Date("UCIAMS 21994 Black bear",10465,25);
R_Date("UCIAMS 5753 Black bear",10485,35);
R_Date("UCIAMS 5756 Black bear",10515,35);
R_Date("UCIAMS 5733 Black bear",10550,35);
Curve("Marine20","marine20.14c");
Delta_R("LocalMarine",257,99);
R_Date("UCIAMS 15163 Salmon",11510,25);
Curve("IntCal20","intcal20.14c");
R_Date("UCIAMS 5755 Black bear",10575,35);
R_Date("UCIAMS 4889 Black bear",10585,45);
R_Date("UCIAMS 15161 Deer",10920,35);
R_Date("UCIAMS 15159 Deer",10935,40);
R_Date("UCIAMS 28003 Deer",10945,25);
R_Date("UCIAMS 23611 Deer",10965,40);
R_Date("UCIAMS 15162 Deer",10990,25);
R_Date("UCIAMS 4892 Deer",11005,45);
R_Date("UCIAMS 15154 Black bear",11030,30);
R_Date("UCIAMS 15160 Deer",11060,30);
R_Date("CAMS 75559 Bear",11150,50);
R_Date("CAMS 79489 Bear",11250,70);
Curve("IntCal20","intcal20.14c");
Curve("Marine20","marine20.14c");
Delta_R("LocalMarine1",257,99);
Mix_Curves("Mixed1","IntCal20","LocalMarine1",30,0);
R_Date("CAMS 75746 Bear",14540,70);
Curve("IntCal20","intcal20.14c");
R_Date("UOC 5713 Caribou",43209,649);
R_Date("CAMS 26265 Richardson Isld.",8700,60);
R_Date("CAMS 26267 Richardson Isld.",8960,60);
R_Date("CAMS 26266 Richardson Isld.",8980,60);
```

```
R_Date("CAMS 26268 Richardson Isld.",9080,60);
R_Date("CAMS 26269 Richardson Isld.",9160,60);
R_Date("CAMS 26270 Richardson Isld.",9220,60);
R_Date("CAMS 76668 Kilgii Gwaay",9230,50);
R_Date("CAMS 79682 Kilgii Gwaay",9260,40);
R_Date("CAMS 39876 Richardson Isld.",9290,50);
R_Date("CAMS 39875 Richardson Isld.",9290,50);
R_Date("CAMS 79684 Kilgii Gwaay",9340,40);
R_Date("CAMS 87642 Kilgii Gwaay",9395,40);
R_Date("CAMS 77248 Kilgii Gwaay",9410,50);
R_Date("CAMS 87641 Kilgii Gwaay",9415,35);
R_Date("CAMS 76666 Kilgii Gwaay",9430,50);
R_Date("CAMS 70704 Kilgii Gwaay",9460,50);
R_Date("UCIAMS 49181 GD2",9485,15);
R_Date("UCIAMS 49183 GD2",9530,15);
R_Date("UCIAMS 12387 GD1",9930,30);
R_Date("UCIAMS 12386 GD1",9980,30);
R_Date("UCIAMS 55085 GD2",10025,45);
R_Date("UCIAMS 31729 GD1",10150,25);
R_Date("UCIAMS 55083 GD2",10205,20);
R_Date("UCIAMS 55084 GD2",10210,20);
R_Date("UCIAMS 49182 GD2",10215,20);
R_Date("UCIAMS 40880 GD2",10220,30);
R_Date("UCIAMS 56932 GD2",10280,30);
R_Date("UCIAMS 40882 GD2",10295,25);
R_Date("UCIAMS 56933 GD2",10530,20);
R_Date("UCIAMS 12388 GD1",10550,25);
R_Date("UCIAMS 4884 K1",10510,35);
R_Date("CAMS 93774 K1",10525,50);
R_Date("UCIAMS 28005 GD1",10615,30);
R_Date("CAMS 93775 K1",10660,40);
R_Date("UCIAMS 4886 K1",10960,35);
```

```
 R_Date("UCIAMS 40881 GD2",11030,25);
 R_Date("UCIAMS 28004 GD1",11665,30);
};
```

Table S4.

Radiocarbon date calibrations and code for the 46 age estimates that comprised the central coast chronology. Source references for each date are in table S1. A: Calibrated radiocarbon age estimates in calendar years befor present (BP), B: Code from OxCal 4.4.4.

**A. Central Coast Subregion (n=46)**	Unmodelled (BP)			
	sigma	median	from_95_4	to_95_4
UCIAMS 56477 NVIsld. Small rodent	44	9602	9684	9542
Beta 10714 NVIsld. Black bear	251	11279	11807	10773
UCIAMS 56478 NVIsld. Small rodent	105	11698	11825	11403
UCIAMS 59635 NVIsld.Vertebrate	89	12561	12697	12210
UCIAMS 41052 NVIsld. Brown bear	50	13042	13101	12926
UCIAMS 118263 NVIsld. Deer mouse	28	13103	13165	13070
UCIAMS 118261 NVIsld. Deer mouse	70	13337	13460	13182
UCIAMS 118259 NVIsld. Deer mouse	65	13368	13488	13237
UCIAMS 41049 NVIsld. Short-faced bear	47	13480	13583	13367
OxA 24005 NVIsld.Short-faced bear	69	13563	13747	13471
UCIAMS 41048 NVIsld. Short-faced bear	72	13638	13753	13512
UCIAMS 118260 NVIsld. Deer mouse	113	13693	14003	13499
UCIAMS 56479 NVIsld. Black bear	104	13802	14018	13609
UCIAMS 73321 NVIsld. Deer mouse	91	13862	14029	13615
TO 5006 NVIsld. Mt. Goat	87	13931	14083	13795
UCIAMS 73320 NVIsld. Bear	82	13991	14089	13809
TO 6072 NVIsld. Mt. Goat	351	14242	14989	13614
CAMS 97342 NVIsld. Mt.Goat	212	14363	14829	14103
UICAMS 118265 NVIsld. Brown bear	191	14432	14832	14184
UICAMS 41050 NVIsld. Brown bear	168	14545	14874	14289
UCIAMS 41051 NVIsld. Brown bear	168	14583	14911	14311
CAMS 88275 NVIsld. Sparrow	224	19659	20101	19187
CAMS 74625 NVIsld. Vole	94	19722	19885	19550
CAMS 102798 NVIsld. Mt. Goat	94	19722	19885	19550
CAMS 88274 NVIsld. Marmot	222	19877	20332	19495
CAMS 97341 NVIsld. Vertebrate	63	20491	20619	20348
CAMS 102797 NVIsld. Vole	97	20660	20845	20490
CAMS 74624 NVIsld. Vole	172	21933	22191	21464
WSU-2141/1978 Bear Cove	168	8876	9265	8593
WAT 519 Namu	212	10094	10505	9674
UICAMS 112263 Triquet Isld.	48	10275	10404	10231
GAK 3244 Namu	301	10324	11069	9682
UCIAMS 61764 Kokish River	48	10552	10655	10438
WAT 452 Namu	225	11081	11607	10601
UCIAMS 163730 Triquet Isld.	22	11242	11312	11201
UCIAMS 118001 Triquet Isld.	89	11357	11607	11262
Beta 109626 Hunter Isld.	112	11361	11683	11238

UCIAMS 184916 Calvert Isld.	34	12671	12712	12618
UCIAMS 163740 Calvert Isld.	42	12714	12760	12620
UCIAMS 169399 Calvert Isld.	51	12878	13054	12767
UCIAMS 163741 Calvert Isld.	28	13142	13227	13098
UCIAMS 142561 Calvert Isld.	43	13178	13293	13111
UCIAMS 169402 Calvert Isld.	39	13242	13305	13175
UCIAMS 149779 Calvert Isld.	49	13307	13414	13187
UCIAMS 163739 Calvert Isld.	45	13313	13415	13236
UCIAMS 179732 Triquet Isld.	282	13920	14807	13486

B. OXCAL CODE

```
Plot(Central Coast Subregion)
{
 R_Date("UCIAMS 56477 NVIsld. Small rodent",8660,30);
 R_Date("Beta 10714 NVIsld. Black bear",9830,140);
 R_Date("UCIAMS 56478 NVIsld.Small rodent",10100,30);
 R_Date("UCIAMS 59635 NVIsld.Vertebrate",10535,50);
 R_Date("UCIAMS 41052 NVIsld.Brown bear",11110,30);
 R_Date("UCIAMS 118263 NVIsld. Deer mouse",11170,30);
 R_Date("UCIAMS 118261 NVIsld. Deer mouse",11460,60);
 R_Date("UCIAMS 118259 NVIsld.Deer mouse",11490,60);
 R_Date("UCIAMS 41049 NVIsld. Short-faced bear",11615,30);
 R_Date("OxA 24005 NVIsld.Short-faced bear",11720,50);
 R_Date("UCIAMS 41048 NVIsld. Short-faced bear",11775,30);
 R_Date("UCIAMS 118260 NVIsld. Deer mouse",11840,90);
 R_Date("UCIAMS 56479 NVIsld. Black bear",11935,40);
 R_Date("UCIAMS 73321 NVIsld. Deer mouse",11960,45);
 R_Date("TO 5006 NVIsld. Mt. Goat",12070,70);
 R_Date("UCIAMS 73320 NVIsld. Bear",12110,45);
 R_Date("TO 6072 NVIsld. Mt. Goat",12200,190);
 R_Date("CAMS 97342 NVIsld. Mt.Goat",12340,50);
 R_Date("UICAMS 118265 NVIsld. Brown bear",12370,35);
 R_Date("UICAMS 41050 NVIsld. Brown bear",12425,35);
 R_Date("UCIAMS 41051 NVIsld. Brown bear",12440,35);
 R_Date("CAMS 88275 NVIsld. Sparrow",16270,170);
 R_Date("CAMS 74625 NVIsld. Vole",16340,60);
 R_Date("CAMS 102798 NVIsld. Mt. Goat",16340,60);
 R_Date("CAMS 88274 NVIsld. Marmot",16460,170);
 R_Date("CAMS 97341 NVIsld. Vertebrate",16965,45);
 R_Date("CAMS 102797 NVIsld. Vole",17100,70);
 R_Date("CAMS 74624 NVIsld. Vole",18010,100);
 R_Date("WSU-2141/1978 Bear Cove",8020,110);
 R_Date("WAT 519 Namu",9000,140);
```

```
 R_Date("UICAMS 112263 Triquet Isld.",9140,25);
 R_Date("GAK 3244 Namu",9140,200);
 R_Date("UCIAMS 61764 Kokish River",9345,25);
 R_Date("WAT 452 Namu",9720,140);
 R_Date("UCIAMS 163730 Triquet Isld.",9845,25);
 R_Date("UCIAMS 118001 Triquet Isld.",9960,25);
 R_Date("Beta 109626 Hunter Isld.",9940,50);
 R_Date("UCIAMS 184916 Calvert Isld.",10625,20);
 R_Date("UCIAMS 163740 Calvert Isld.",10720,60);
 R_Date("UCIAMS 169399 Calvert Isld.",10980,25);
 R_Date("UCIAMS 163741 Calvert Isld.",11260,25);
 R_Date("UCIAMS 142561 Calvert Isld.",11295,30);
 R_Date("UCIAMS 169402 Calvert Isld.",11365,25);
 R_Date("UCIAMS 149779 Calvert Isld.",11435,30);
 R_Date("UCIAMS 163739 Calvert Isld.",11440,25);
 R_Date("UCIAMS 179732 Triquet Isld.",12010,180);
};
```

Table S5.

Radiocarbon date calibrations and code for the 47 age estimates that comprised the south coast chronology. Source references for each date are in table S1. A: Calibrated radiocarbon age estimates in calendar years befor present (BP), B: Code from OxCal 4.4.4.

**A. South Coast Subregion (n=47)**	Unmodelled (BP)			Modelled (BP)		
	median	from_95_	to_95_4	median	from_95_	to_95_4
Beta 199434 Whidbey Isld. Vole	9281	9426	9128	9281	9426	9127
GX 25892 AMS Whidbey Isld. Deer	9927	10158	9700	9927	10160	9701
Beta 231169 Orcas Isld. Bison	12750	12840	12690	12751	12840	12690
Beta 231168 Orcas Isld. Bison	13106	13226	12925	13106	13225	12925
SFU-BG-1 Victoria Bison	13617	13981	13351	13616	13979	13349
Beta 216160 Orcas Isld. Bison	13625	13773	13490	13623	13775	13490
UCIAMS 11677 Manis Mastodon	13744	13973	13603	13745	13972	13603
UCIAMS 11350 Manis Mastodon	13891	14023	13777	13893	14024	13776
UCIAMS 12046 Manis Mastodon	13891	14023	13777	13889	14024	13778
Combined Manis Mastodon	13924	14016	13785	13922	14016	13785
UCIAMS 29113 Manis Mastodon	13909	14023	13790	13910	14023	13790
UCIAMS 53549 Orcas Isld. Bison	13911	14021	13791	13912	14021	13791
Marine20						
LocalMarine	267	163	371	266	161	370.5
Beta 115202 Courtney Sea lion	13658	13954	13420	13658	13957	13416
IntCal20						
GSC 2829 Victoria Mammoth	20544	21145	19900	20543	21146	19900
UCIAMS 56480 Victoria Short-faced bear	27106	27319	26470	27105	27321	26470
GaK 4866 Glenrose Cannery Vancouver	9071	9555	8418	9072	9585	8422
UCIAMS 179749 Quadra/Yeatman Bay 1	9450	9488	9320	9450	9489	9320
UCIAMS 179748 Quadra/Yeatman Bay 2	9610	9683	9548	9611	9684	9548
Beta 204766 Stave/DhRn-29	9544	9677	9487	9545	9676	9487
Wk 19455 Stave/DhRn-21	9645	9888	9542	9645	9888	9542
UCIAMS 175362 Quadra/Lactarius	9816	10110	9690	9815	10110	9690
UCIAMS 175360 Quadra/Lactarius	9848	10119	9700	9847	10120	9700

UCIAMS 175363 Quadra/Lactarius	10003	10153	9781	10003	10153	9782
UCIAMS 169391 Quadra/Lactarius	10024	10160	9895	10024	10160	9896
UCIAMS 193675 Quadra/Yeatman Bay 1	10056	10194	9915	10056	10194	9915
Beta 194838 Stave/DhRn-29	10062	10227	9907	10062	10227	9907
Wk 19454 Stave/DhRn-16	10175	10242	9915	10175	10242	9916
Wk 19449 Stave/DhRo-11	10230	10283	10186	10230	10283	10185
Beta 245384 Stave/DhRn-29	10352	10499	10240	10352	10499	10240
Beta 191869 Stave/DhRn-29	10377	10500	10252	10378	10500	10252
UCIAMS 193680 Quadra/Yeatman Bay 1	10391	10510	10250	10390	10549	10249
Beta 194839 Stave/DhRn-29	10417	10569	10253	10417	10569	10253
UCIAMS 193678 Quadra/Yeatman Bay 1	10516	10578	10420	10516	10578	10421
UCIAMS 196775 Quadra/Yeatman Bay 1	10770	11069	10603	10769	11070	10604
UCIAMS 196776 Quadra/Yeatman Bay 1	10941	11073	10710	10943	11073	10711
UCIAMS 193679 Quadra/Yeatman Bay 1	10926	11147	10780	10926	11147	10780
Beta 355417 Stave/DhRn-18	11212	11261	11161	11212	11261	11160
Beta 355419 Stave/DhRn-18	11331	11610	11225	11331	11610	11224
Beta 355416 Stave/DhRn-18	11519	11744	11280	11519	11744	11280
Beta 226980 Stave/DhRn-29	11786	11940	11508	11785	11939	11508
Beta 245383 Stave/DhRo-16	11880	12002	11744	11879	12002	11744
Beta 249280 Stave/DhRo-16	12051	12458	11827	12050	12458	11828
Beta 241999 Stave/DhRn-29	12243	12473	12002	12243	12472	12003
UCIAMS 193684 Quadra/EbSh-81	12722	12823	12618	12722	12824	12618
UCIAMS 193676 Quadra/Yeatman Bay 1	12851	13058	12750	12852	13058	12749
Clovis						
Clovis start	13008	13096	12903	13007	13095	12903
Clovis end	12736	12755	12719	12736	12755	12718
Manis mastodon combined	13924	14016	13785	13920	14015	13785
UCIAMS 53549 Bison Orcas Isld./Ayer	13911	14021	13791	13911	14021	13791

B. OXCAL CODE

Plot(South Coast Subregion)

```
{
R_Date("Beta 199434 Whidbey Isld. Vole",8280,40);
R_Date("GX 25892 AMS Whidbey Isld. Deer",8840,50);
R_Date("Beta 231169 Orcas Isld. Bison",10800,60);
R_Date("Beta 231168 Orcas Isld. Bison",11180,60);
R_Date("SFU-BG-1 Victoria Bison",11750,110);
R_Date("Beta 216160 Orcas Isld. Bison",11760,70);
R_Date("UCIAMS 11677 Manis Mastodon",11890,35);
R_Date("UCIAMS 11350 Manis Mastodon",11975,35);
R_Date("UCIAMS 12046 Manis Mastodon",11975,35);
R_Date("Combined Manis Mastodon",11969,18);
R_Date("UCIAMS 29113 Manis Mastodon",11990,30);
R_Date("UCIAMS 53549 Orcas Isld. Bison",11990,25);
Curve("Marine20","marine20.14c");
Delta_R("LocalMarine",267,52);
R_Date("Beta 115202 Courtney Sea lion",12570,70);
Curve("IntCal20","intcal20.14c");
R_Date("GSC 2829 Victoria Mammoth",17000,240);
R_Date("UCIAMS 56480 Victoria Short-faced bear",22750,140);
R_Date("GaK 4866 Glenrose Cannery Vancouver",8150,250);
R_Date("UCIAMS 179749 Quadra/Yeatman Bay 1",8405,20);
R_Date("UCIAMS 179748 Quadra/Yeatman Bay 2",8680,20);
R_Date("Beta 204766 Stave/DhRn-29",8590,40);
R_Date("Wk 19455 Stave/DhRn-21",8702,43);
R_Date("UCIAMS 175362 Quadra/Lactarius",8800,20);
R_Date("UCIAMS 175360 Quadra/Lactarius",8820,25);
R_Date("UCIAMS 175363 Quadra/Lactarius",8855,20);
R_Date("UCIAMS 169391 Quadra/Lactarius",8875,20);
R_Date("UCIAMS 193675 Quadra/Yeatman Bay 1",8935,20);
R_Date("Beta 194838 Stave/DhRn-29",8950,50);
R_Date("Wk 19454 Stave/DhRn-16",8990,46);
R_Date("Wk 19449 Stave/DhRo-11",9075,34);
```

```
 R_Date("Beta 245384 Stave/DhRn-29",9190,50);
 R_Date("Beta 191869 Stave/DhRn-29",9220,40);
 R_Date("UCIAMS 193680 Quadra/Yeatman Bay 1",9230,45);
 R_Date("Beta 194839 Stave/DhRn-29",9250,60);
 R_Date("UCIAMS 193678 Quadra/Yeatman Bay 1",9305,20);
 R_Date("UCIAMS 196775 Quadra/Yeatman Bay 1",9505,30);
 R_Date("UCIAMS 196776 Quadra/Yeatman Bay 1",9540,25);
 R_Date("UCIAMS 193679 Quadra/Yeatman Bay 1",9605,20);
 R_Date("Beta 355417 Stave/DhRn-18",9780,40);
 R_Date("Beta 355419 Stave/DhRn-18",9920,50);
 R_Date("Beta 355416 Stave/DhRn-18",10020,50);
 R_Date("Beta 226980 Stave/DhRn-29",10150,40);
 R_Date("Beta 245383 Stave/DhRo-16",10210,40);
 R_Date("Beta 249280 Stave/DhRo-16",10290,50);
 R_Date("Beta 241999 Stave/DhRn-29",10370,40);
 R_Date("UCIAMS 193684 Quadra/EbSh-81",10740,70);
 R_Date("UCIAMS 193676 Quadra/Yeatman Bay 1",10940,60);
 Phase("Clovis")
 {
  R_Date("Clovis start",11080,40);
  R_Date("Clovis end",10765,25);
 };
 R_Date("Manis mastodon combined",11969,18);
 R_Date("UCIAMS 53549 Bison Orcas Isld./Ayer",11990,25);
};
```

Table S6.

Marine reservoir offsets in marine samples from the north coast (southeast Alaska and Haida Gwaii) and the south coast were calculated using the following ΔR values from the 14CHRON Marine20 marine radiocarbon reservoir database at http://calib.org/marine/ (*106*).

North Coast (southeast Alaska and Haida Gwaii)

**14CHRON Map#**	**Lon**	**Lat**	**ΔR**	**ΔR Error**	**Reference**	**Locality**
932	-146.33	59.43	217	50	*153*	Middleton Is., AK
958	-145.7	60.57	289	20	*153*	Orca, AK
931	-135.5	57	248	60	*153*	Sitka Sound, AK
934	-136.35	58.76	301	60	*153*	Glacier Bay, AK
939	-135	57.5	366	50	*153*	Chatham, AK
940	-135	57.5	306	50	*153*	Chatham, AK
935	-134.1	57.1	511	60	*153*	Admiralty Is., AK
937	-134.1	57.1	381	70	*153*	Admiralty Is., AK
933	-154	57.7	177	50	*153*	Kodiak Is., AK
938	-132.42	53.3	76	50	*153*	Graham Is., BC
941	-132.42	53.3	176	50	*153*	Graham Is., BC
952	-132.1	54.02	247	50	*153*	Masset, BC
950	-132	53.23	73	50	*153*	Skidegate, BC
951	-132	53.23	43	40	*153*	Skidegate, BC
936	-132.85	57.3	441	50	*153*	Thomas Bay, AK
942	-132.85	57.3	311	60	*153*	Thomas Bay, AK
943	-132.85	57.3	371	60	*153*	Thomas Bay, AK
2206	-130.693	54.198	273	67	*108*	Hecate Strait
2205	-130.693	54.198	303	66	*108*	Hecate Strait
2204	-130.693	54.198	276	68	*108*	Hecate Strait
2207	-130.693	54.198	286	65	*108*	Hecate Strait
2208	-130.693	54.198	259	68	*108*	Hecate Strait
2209	-130.693	54.198	250	67	*108*	Hecate Strait
2210	-130.693	54.198	236	69	*108*	Hecate Strait
2211	-130.693	54.198	254	65	*108*	Hecate Strait
2212	-130.693	54.198	243	66	*108*	Hecate Strait
2213	-130.693	54.198	269	67	*108*	Hecate Strait
2214	-130.693	54.198	241	66	*108*	Hecate Strait
2215	-130.693	54.198	221	66	*108*	Hecate Strait
2216	-130.693	54.198	253	64	*108*	Hecate Strait
2217	-130.693	54.198	254	63	*108*	Hecate Strait
2218	-130.693	54.198	226	64	*108*	Hecate Strait
**AVERAGE ΔR**			**257**	**99**		

South Coast - Pacific Inner Passage (Georgia Strait / Puget Sound)

919	-124.82	49.93	387	50	*153*	Savary Is., BC
923	-124.2	49.27	247	50	*153*	Nanoose Bay, BC
921	-124.17	49.49	257	50	*153*	Comox, BC
149	-124	48.4	245	50	*154*	Sooke, British Columbia, Canada
922	-123.95	49.2	282	50	*153*	Departure Bay, BC
916	-124	49.5	355	40	*153*	Georgia Strait, BC
925	-123.72	48.38	199	50	*153*	Sooke, BC
930	-123.72	48.38	289	20	*153*	Sooke, BC
924	-123.7	48.92	257	50	*153*	Chemainus Bay, BC
928	-123.7	48.92	197	70	*153*	Chemainus Bay, BC
150	-123.43	48.43	146	50	*154*	Esquimalt, British Columbia, Canada
920	-123.37	48.43	271	50	*153*	Victoria, BC
929	-123.37	48.43	236	20	*153*	Victoria, BC
926	-123.2	49.3	255	40	*153*	Burrard Inlet, BC
918	-123.2	48.1	256	50	*153*	Port Crescent, WA
917	-123.1	48.2	265	50	*153*	San Juans, WA
148	-123	48.6	199	50	*154*	Orcas Island, Washington, USA
151	-123	48.6	344	30	*154*	Orcas Island, Washington, USA
927	-122.5	48	233	40	*153*	Puget Sound, WA
**AVERAGE ΔR**			**267**	**52**		

Table S7.

Bone samples previously reported as mixed terrestrial-marine feeders were assigned a percent marine amount based on δ^{13}C and δ^{15}N values. Published isotope values on bone samples were preferred. Where δ^{15}N was not published with a radiocarbon date, I estimated this value based on published values that best match the spatial and temporal resources available to the mixed feeders in our study. Bone collagen-collagen trophic enrichment factor of δ^{13}C 1.0 ± 0.3‰ and δ^{15}N 4.2 ± 1.4‰ based on data from archaeological sites (*111*). The FRUITS 3.0 Beta program (*109*) was used to model the proportional contributions of terrestrial and marine food sources to the value of the δ^{13}C and δ^{15}N isotopes in the bone samples.

Values used to estimate %Marine in the diets of mixed feeders from Haida Gwaii and southeast Alaska subregions with FRUITS 3.0 Beta.

**Target Haida Gwaii (post-glacial late Pleistocene)**								
	δC^{13}	error	δN^{15}	error	reference		%Marine (Salmon)	error
1 Brown bear UCIAMS15156	–15.6	0	16.4	0	*83*		0.78	0.004
2 Brown bear UCIAMS41042	–16.1	0	15	0	*83*		0.65	0.003
3 Brown bear UCIAMS41043	–16.5	0	14.2	0	*83*		0.39	0.004
**AVERAGE brown bear**	**–15.6**	**0.37**	**15.2**	**0.91**	*83*			
**Source Haida Gwaii (post-glacial late Pleistoce**	δC^{13}	error	δN^{15}	error	reference			
1 Salmon UCIAMS15163	–16.6	0	13.8	0	*83*			
2 Salmon UCIAMS15164	–15.5	0	14.6	0	*83*			
3 Salmon UCIAMS5750	–14.8	0	15	0	*83*			
4 Salmon UCIAMS5754	–15.4	0	15.6	0	*83*			
5 Salmon UCIAMS15165	–14.7	0	15.7	0	*83*			
**AVERAGE Salmon**	**–15.4**	**0.68**	**14.94**	**0.7**				
							**Terrestrial combined (deer and black bear values)**	
1 Deer UCIAMS4892	–20.7	0	2.2	0	*83*		δC^{13}	δN^{15}
2 Deer UCIAMS23611	–21.6	0	4.4	0	*83*		20.7	2.2
3 Deer UCIAMS15159	–21.4	0	3.4	0	*83*		21.6	4.4
4 Deer UCIAMS15162	–22.1	0	6	0	*83*		21.4	3.4
5 Deer UCIAMS15161	–22.4	0	6.1	0	*83*		22.1	6
6 Deer UCIAMS28003	–19.5	0	6.4	0	*83*		22.4	6.1
**AVERAGE Deer**	**–21.28**	**0.96**	**4.75**	**1.56**			19.5	6.4
							20.6	0.8
1 Black bear UCIAMS5756	–20.6	0	0.8	0	*83*		19.1	6.9
2 Black bear UCIAMS21994	–19.1	0	6.9	0	*83*		21.3	2.7
3 Black bear UCIAMS5755	–21.3	0	2.7	0	*83*		21.7	2.3
4 Black bear UCIAMS4889	–21.7	0	2.3	0	*83*		21.5	2.4
5 Black bear UCIAMS5733	–21.5	0	2.4	0	*83*		20.2	3.8
6 Black bear UCIAMS15154	–20.2	0	3.8	0	*83*	**AVERAGE**	**21.01**	**3.95**
**AVERAGE Black bear**	**–20.73**	**0.9**	**3.15**	**1.9**		**SD**	**0.97**	**1.91**

**Source SEA (post-glacial late Pleistocene)**								
	δC^{13}	error	n	reference	δN^{15}	error	n	reference
1 Brown bear	–17.5	3.6	17	*77*	15.2	1.11	3	*83*

		δC13	error	n	reference	δN15	error	n	reference
2	Arctic fox	–15.5	7.1	8	*77*	8	0.6	13	*143* (modern bone)
3	Red fox	–15.2	4.7	3	*77*	11.93	3.4	11	*144*

Source SEA (post-glacial late Pleistocene)

		δC^{13}	error	n	reference	δN^{15}	error	n	reference
	Terrestrial								
1	Beaver	–25.7	0	1	*77*	3.03	0.04	4	*145* (modern bone)
2	Deer	–24.6	3.2	8	*77*	4.75	1.56	6	*83*
3	Hoary marmot	–22.4	3.5	7	*77*	1.15	0.05	2	*146* & this publication
4	Black bear	–20.7	5.2	44	*77*	3.15	1.9	6	*83*
5	Sagia antelope	–19.4	2.2	3	*77*	5.5	2.4	2	*147*
6	Caribou	–19.0	1.2	4	*77*	3.27	1.12	10	*148*
	**AVERAGE terrestrial**	**–21.2582**	**4.74**	**67**		**3.52**	**1.64**	**30**	
	Marine								
7	Cetacea (great whale)	–14.5	0	1	*77*	15.41	1.5	9	*149*
8	Sea birds	–14.1	3.5	6	*77*	16.3	0.6	3	*150*
9	Steller sea lion	–13.6	1.1	3	*77*	16.6	1.1	11	*149*
10	Marine fishes	–13.5	5	3	*77*	15.58	1.69	29	*149*
11	Ringed seal	–13.2	4.7	25	*77*	17.71	0.79	5	*149*
12	Harbor seal	–12.5	4.7	4	*77*	17.9	1.1	9	*151*
13	Sea otter	–11.6	2.6	3	*77*	13.2	0.6	10	*152*
	**AVERAGE marine**	**–18.0312**	**5.9741**	**179**		**15.83**	**1.9**	**74**	

Fractionation values

$\delta^{13}C$ 1.0 error 0.3‰ (*111*)

$\delta^{15}N$ 4.2 error 1.4‰ (*111*)
